# Anterior-posterior constraint on Hedgehog signaling by *hhip* in teleost fin elaboration

**DOI:** 10.1242/dev.202526

**Published:** 2024-11-18

**Authors:** Yoshitaka Tanaka, Shun Okayama, Kohei Urakawa, Hidehiro Kudoh, Satoshi Ansai, Gembu Abe, Koji Tamura

**Affiliations:** ^1^ Department of Ecological Developmental Adaptability Life Sciences, Graduate School of Life Sciences, Tohoku University, Sendai 980-8578, Japan; ^2^ Laboratory of Genome Editing Breeding, Graduate School of Agriculture, Kyoto University, Kyoto 606-8507, Japan; ^3^ Department of Developmental Biology, Integrated Medical Sciences, Graduate School of Medical Sciences, Tottori University, Yonago 683-8503, Japan; ^4^ Department of Integrative Life Sciences, Graduate School of Life Sciences, Tohoku University, Sendai 980-8577, Japan; ^5^ Division of Developmental Biology, Department of Functional Morphology, School of Life Science, Faculty of Medicine, Tottori University, Yonago 683-8503, Japan

**Keywords:** Evo-devo, Fin development, Hhip, Shh, Teleost

## Abstract

Pectoral fins, the anterior paired fins in fish, have enhanced maneuvering abilities due to morphological changes. Teleosts have fewer radial bones in their pectoral fins than basal species, resulting in more-elaborate fins. The mechanism behind this radial constraint change in teleosts is unclear. Here, we found that mutations in *hhip*, which encodes an antagonist of Hedgehog signaling, led to an increase in radial bones in a localized region. Expression of the Shh genes, encoding ligands of Hedgehog signaling, coincided with notable *hhip* expression specifically during early development. We suggest that a negative feedback effect of Hedgehog signaling by *hhip* regulates the constraint of the pectoral fin in zebrafish. Additionally, re-analysis of *hhip*-related gene expression data in zebrafish and basal species revealed that the notable *hhip* expression during early development is a characteristic of zebrafish that is not observed in basal species. Region-specific expression of Hox13 genes in the zebrafish pectoral fin indicated that the median region, analogous to the region with abundant radials in basal species, is expanded in *hhip^−/−^* zebrafish. These data underscore potential morphological evolution through constrained diversity.

## Teaser

**Summary:** Mutation of *hhip* in zebrafish induces abundant radial bones of pectoral fins, analogous to those of basal fishes, suggesting the importance of Hedgehog signaling suppression by *hhip* in teleosts.

## INTRODUCTION

Fins in fishes are a product of evolutionary adaptation and enhance diverse hydrodynamic maneuvers, including speed adjustments, hovering, and directional changes in the water. It is particularly noteworthy that pectoral fins, the anterior paired fins, have undergone morphological transformations in actinopterygians, resulting in a transition from their ancestral state to a derived form within the skeletal framework. In the ancestral state, pectoral fins possess a large number of radial bones, as observed in basal species, such as chondrichthyans, some basal actinopterygians, placoderms, and acanthodians (Coates, 1994, 2003; Davis et al., 2004; Onimaru et al., 2015; Starck, 1979; Tanaka, 2018). The pectoral fins of basal species are composed of multiple basal radials, comprising the tribasal bones (propterygium, mesopterygium, and metapterygium) (Gegenbaur, 1865; Onimaru et al., 2015; Tanaka, 2018). These fishes use wide pectoral fins for steady swimming or vertical directional changes (Wilga and Lauder, 1999, 2000). By contrast, pectoral fins in teleosts, derived fishes in actinopterygians, have lost these ancestral characteristics and have only a limited number of radials remaining (Grandel and Schulte-Merker, 1998; Tanaka et al., 2022; Woltering et al., 2018). While almost all teleosts have only four radials, some (e.g. Osteoglossiformes, Siluriformes, Stomiiformes) have fewer than three radials and only a few others have more than four radials (Tanaka et al., 2022). As a consequence of the radial constraint, teleosts have elaborated their pectoral fins into compact ones for speed adjustment, hovering, or horizontal directional changes (Drucker and Lauder, 2002, 2003).

Elaboration of pectoral fins in teleosts has occurred along the anterior-posterior (AP) axis. In the development of vertebrate appendages, Hedgehog signaling regulates skeletal morphology along the AP axis by promoting cell proliferation and patterning/differentiation of the appendicular skeleton. *Sonic hedgehog* (*Shh*) is one of the genes encoding a Hedgehog signaling ligand, and it regulates the number of skeletal components along the AP axis in paired appendages (Riddle et al., 1993). In limbs, which are paired appendages in tetrapods (amphibians and amniotes), and homologous to paired fins, disruption or mutation of a limb-specific *Shh* enhancer results in digit loss or polydactyly along the AP axis (Lettice et al., 2003, 2017; Sagai et al., 2005). In paired fins, disruption of *Shh* expression also causes severe defects in paired fin skeletons (Letelier et al., 2018; Neumann et al., 1999). Therefore, it is plausible that *Shh* regulation along the AP axis is involved in elaboration of pectoral fins in teleosts.

Based on previous reports, we hypothesized that repression of Shh signaling in teleost pectoral fins moderately reduces the number of pectoral fin radials. To investigate whether repression of Shh signaling affects the number of radials, we focused on repressive mediators of the Hedgehog signaling pathway. We generated knockout models of repressive mediators of Hedgehog signaling in zebrafish and studied their development and the changes in their pectoral fin skeletons. We found that overactivation of Hedgehog signaling by mutation of a repressive mediator increased the number of pectoral fin radials. Our results suggest that the AP constraint on Hedgehog signaling by the repressive mediator has reduced pectoral fin radials compared to those of basal species and resulted in pectoral fin elaboration in teleosts.

## RESULTS

### Overactivation of Hedgehog signaling by *hhip* mutations induces enlargement of endochondral components in pectoral and other fin skeletons

As candidate genes involved in the repression of Shh signaling, we examined two genes mediating the repression of Hedgehog signaling, *hedgehog interacting protein* (*hhip*) and *GLI family zinc finger 3* (*gli3*). Zebrafish with *hhip* mutations are known to exhibit an increase in the number of fin rays and an enlarged pectoral fin primordia, but the endochondral components in paired fin skeletons have not been characterized (Koudijs et al., 2005; van Eeden et al., 1996). *gli3* mutant medaka have been reported to exhibit an increase in the number of paired fin radials (Letelier et al., 2021), but the fin radial phenotype in *gli3* mutant zebrafish has not yet been reported. Using the CRISPR-Cas9 genome-editing approach in zebrafish, we introduced a mutation in exon 4 of *hhip* (Fig. S1A) and in exon 5 of *gli3* (Fig. S2A) to replicate previously reported mutants (Koudijs et al., 2005; Letelier et al., 2021). We isolated two *hhip* mutants (*hhip^H215RfsX1^*, *hhip^F242SfsX8^*; Fig. S1A) and one *gli3* mutant with a 122-bp deletion in exon 5 (*gli3*^Δ^; Fig. S2A). *hhip^−/−^* zebrafish showed an enlargement of fin skeletons (Fig. S1B-E), while *gli3*^Δ*/*Δ^ zebrafish did not show any radial fin abnormalities (Fig. S2B-E). Previous studies on *gli3* in zebrafish have also not reported any phenotypes in fin radials (Devine et al., 2009; Wei et al., 2023 preprint). In *hhip^−/−^* zebrafish, the endochondral disk of the pectoral fin was elongated along the AP axis compared to the proximal-distal (PD) axis (Fig. S1B-E). Therefore, we focused on the fin skeletons of adult *hhip* mutants.

**Fig. 1.**
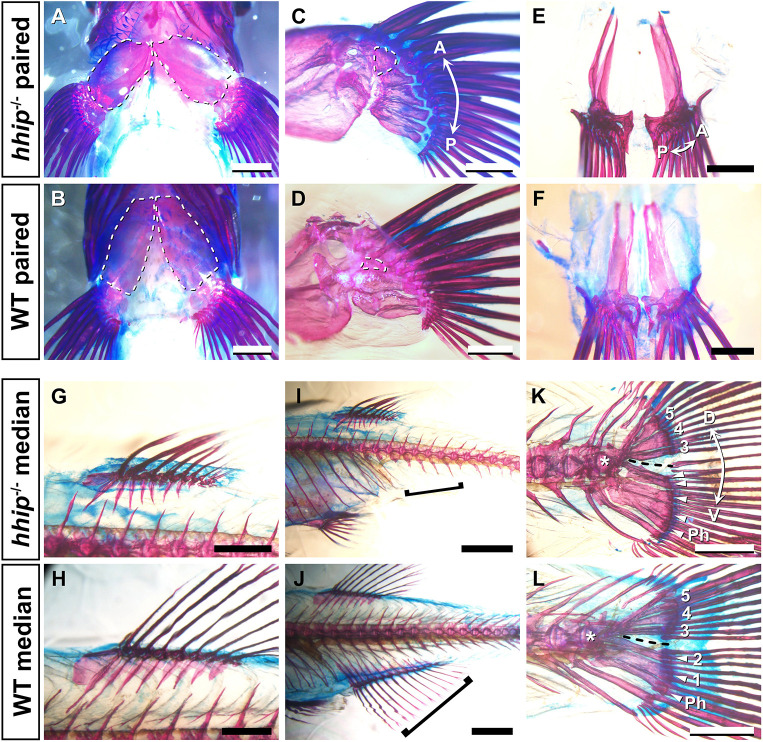
**Paired and median fin skeletons in *hhip^−/−^* zebrafish.** (A,B) Ventral views of the pectoral fin skeleton of *hhip^−/−^* (A) and WT (B) zebrafish. White dashed lines indicate the pectoral girdles. (C,D) Medial views of the pectoral fin skeleton of *hhip^−/−^* (C) and WT (D) zebrafish. White dashed lines indicate the first proximal radial (PR1). (E,F) Pelvic fin skeletons of *hhip^−/−^* (E) and WT (F) zebrafish. (G,H) Dorsal fin skeletons of *hhip^−/−^* (G) and WT (H) zebrafish. (I,J) Anal fin skeletons of *hhip^−/−^* (I) and WT (J) zebrafish. Black brackets indicate the post-anal region where the anal fin is formed. (K,L) Caudal fin skeletons of *hhip^−/−^* (K) and WT (L) zebrafish. Black dashed lines represent the hypural diastema, and the white arrowheads indicate hypurals in the region ventral to the hypural diastema. Numbers indicate the first to fifth hypurals. The white asterisk marks the first ural vertebra. Ph, parhypural. All observations were performed on five *hhip^−/−^* and five WT fish. Double arrows indicate the anterior (A)-posterior (P) axis and the dorsal (D)-ventral (V) axis. Scale bars: 500 µm (C-F); 1 mm (A,B,G,H,K,L); 2 mm (I,J).

In the pectoral fin of *hhip* mutants, the position of the pectoral girdle was shifted laterally compared to that of wild-type (WT) zebrafish (Fig. 1A,B), and the pectoral fin skeletons of *hhip* mutants showed an increased number of radials compared to WT zebrafish (Fig. 1C,D; Fig. S1F). The pelvic fin of *hhip* mutants showed no superficial differences in the endochondral skeletons compared with WT (Fig. 1E,F). The *hhip* mutation also affected median fin skeletons: dorsal, anal, and caudal (Fig. 1G-L). The dorsal fin of *hhip* mutants showed an increased number of radials compared to WT zebrafish (Fig. 1G,H; Fig. S1H, Fig. S3A,C). In contrast, the anal fin skeleton was completely absent in *hhip* mutants (Fig. 1I,J; Fig. S3B,D). In the caudal fin of *hhip* mutants, the number of hypurals, bones formed ventral to the ural centra and supporting the caudal fin, was increased compared to WT zebrafish (Fig. 1K,L). The number of the hypurals in the region ventral to the hypural diastema, the gap between the second and third hypurals (Fig. 1K,L, black dashed line), increased from two to five (Fig. 1K). These results indicate that *hhip^−/−^* zebrafish have an increased number of radials or hypurals in almost all fin skeletons.

Next, to identify the role of *hhip* in fin development, we examined the expression pattern of *hhip*. The expression pattern of *hhip* in the early stages has been previously reported: *hhip* is expressed in the entire pectoral fin bud at 48 h post-fertilization (hpf) (Koudijs et al., 2005). We observed that *hhip* was expressed only at a low level in the posterior margin of the pectoral fin after 5 days post-fertilization (dpf) (Fig. S4B,C). In median fins, *hhip* was highly expressed in the fin rays, but not in the endochondral regions (Fig. S4D). In pelvic fin buds, *hhip* was strongly expressed (Fig. S4E), similar to what was observed in pectoral fin buds at 48 hpf. These results suggest that *hhip* functions specifically during early development of the paired fins in zebrafish.

We then examined whether Hedgehog signaling is overactivated in *hhip* mutants. As a marker gene of Hedgehog signaling activity, one of the Hedgehog downstream target genes, *ptch1*, was selected for the study (Chiang et al., 2001; Matsubara et al., 2016). In WT, *ptch1* was expressed in the posterior margin of pectoral fin buds (Fig. S4F, black arrowhead). Interestingly, in *hhip* mutants, *ptch1* expression was not only observed in the posterior region but also expanded to the anterior region (Fig. S4G). Particularly, *ptch1* expression was high in the anterior and posterior margins (Fig. S4G, black arrowheads). Therefore, *ptch1* expression in *hhip* mutants expanded more anteriorly than in WT. Considering that phenotypes of *hhip^−/−^* zebrafish can be rescued by Hedgehog-repressive treatment (Koudijs et al., 2005), these data indicate that Hedgehog signaling overactivation by *hhip* mutations induces the enlargement of fin skeletons.

### Enlargement of the pectoral skeleton and other fin skeletons in *hhip* mutants occurs in a region-specific manner

**Fig. 2.**
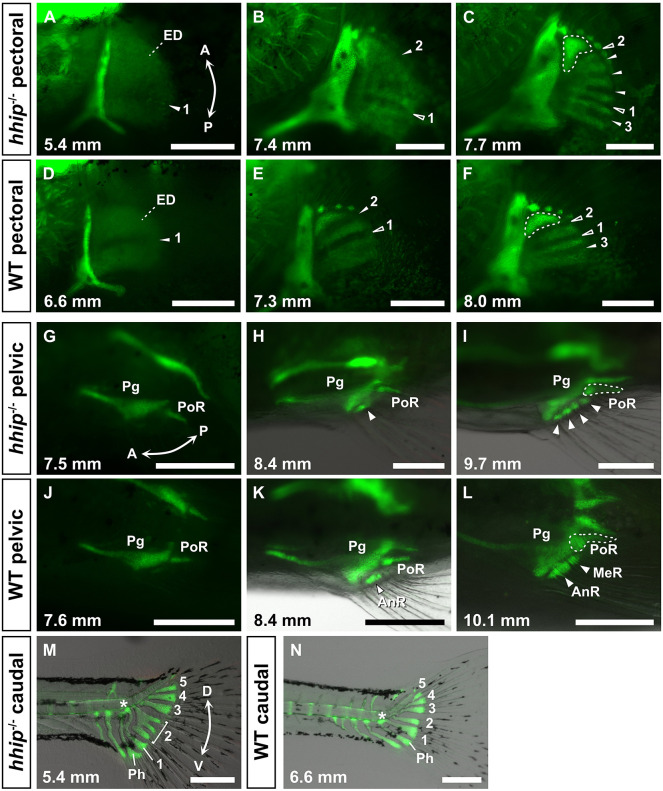
**Developmental processes of the fin skeletons in *hhip****^−/−^* **zebrafish.** (A-F) Pectoral fin development in *hhip^−/−^* (A-C) and WT (D-F) zebrafish. Filled and unfilled white arrowheads indicate new and previous subdivisions, respectively, and numbers mark the predicted first to third subdivisions. White dashed lines indicate the first proximal radial (PR1). (G-L) Pelvic fin development in *hhip^−/−^* (G-I) and WT (J-L) zebrafish. White arrowheads indicate the radial bones. AnR, anterior large radial; MeR, medial small radial; Pg, pelvic girdle; PoR, posterior elongated radial. (M,N) Caudal fin development in *hhip^−/−^* (M) and WT (N) zebrafish. Numbers indicate the first to fifth hypurals. The white asterisk marks the first ural vertebra. Ph, parhypural. These observations were conducted on more than ten larvae at each developmental stage. Green fluorescence indicates the endochondral skeleton marked by *col2a1a:EGFP*. Double arrows indicate the anterior (A)-posterior (P) axis and the dorsal (D)-ventral (V) axis. The standard length of individuals (in mm) is shown in the bottom left of each panel. Scale bars: 250 µm.

Although *hhip^−/−^* zebrafish exhibited an increase in the number of fin skeletal elements, adult fin skeletons were often fused and unrecognizable. To demonstrate how fin radials form and enlarge in detail, we examined fin skeletons during the development of *hhip^−/−^* zebrafish (Fig. 2).

In the development of WT pectoral fins, radials are formed by the subdivisions of an endochondral disk, a large cartilage plate (Dewit et al., 2011; Grandel and Schulte-Merker, 1998). The first subdivision occurs in the middle of the endochondral disk, and the second and third subdivisions occur in the anterior and posterior halves, respectively (Fig. 2D-F). As a result, there are four radials in the WT pectoral fin skeleton. In *hhip^−/−^* zebrafish, the first subdivision shifted posteriorly (Fig. 2A, number 1), and the second subdivision occurred in the anterior third (Fig. 2B, number 2). Subsequently, other subdivisions occurred (Fig. 2C, arrowheads without numbers). The posterior half was further divided only once (Fig. 2C, number 3). These observations indicate that the three featured subdivisions in *hhip^−/−^* zebrafish are equivalent to the first to third subdivisions in WT zebrafish. The first subdivision in both WT and *hhip^−/−^* zebrafish occurred much earlier than the other subdivisions (Fig. 2A,D), suggesting that these subdivisions are equivalent (Fig. 2D, number 1). The second subdivision in WT formed the first proximal radial (PR1; Fig. 2F, dashed line), which has a curved shape different from the other proximal radials. The second subdivision in *hhip^−/−^* zebrafish also formed the PR1 (Fig. 2C, dashed line). Therefore, the second subdivision in *hhip^−/−^* zebrafish appears to be equivalent to the second subdivision in WT (Fig. 2E, number 2). Since both WT and *hhip^−/−^* zebrafish formed two radials from the posterior half of the endochondral disk by a certain subdivision, the subdivision in *hhip^−/−^* zebrafish also appears to be equivalent to the third subdivision in WT (Fig. 2F, number 3). Comparing the subdivision sequence in *hhip^−/−^* and WT zebrafish, the position where proximal radials increased in *hhip^−/−^* zebrafish appears to be in the anterior-median region, namely the second proximal radial of the WT pectoral fins.

The pelvic and caudal fin skeletons in *hhip^−/−^* zebrafish showed abnormalies during development (Fig. 2G-N). In the development of WT pelvic fins, the skeletons possessed three radials (Fig. 2J-L): an anterior large radial (AnR), a medial small radial (MeR), and a posterior elongated radial (PoR). The anterior radials in *hhip^−/−^* zebrafish were as small as the MeR in WT, but not as large as the AnR in WT (Fig. 2G-I). In *hhip^−/−^* zebrafish, although the radials were fused and indistinguishable (Fig. 1E), the number of radials increased to more than four (Fig. 2I; Fig. S1G). The most posterior radial was equivalent to the PoR in WT, suggesting that the *hhip* mutation did not affect the PoR (Fig. 2G-I). Based on these observations, the increase in pelvic radials in *hhip^−/−^* zebrafish occurred in the anterior region that is equivalent to the AnR and MeR in WT zebrafish. In the development of the WT caudal fin, the parhypural and first hypural were formed ventral to the first ural vertebra (Fig. 2N, asterisk), and the second hypural was generated ventral to the second ural vertebra (Desvignes et al., 2018) (Fig. 2N, number 2). In *hhip^−/−^* zebrafish, the primordium of the second hypural was branched, and three hypurals were consequently formed (Fig. 2M, number 2), suggesting that hypurals increased in the ventral region equivalent to the second hypural in WT zebrafish. Therefore, the paired and caudal fins of *hhip^−/−^* zebrafish had a region-specific increase of radials or hypurals, respectively.

### Duplicated Shh genes in zebrafish show a different expression pattern during paired fin developments

**Fig. 3.**
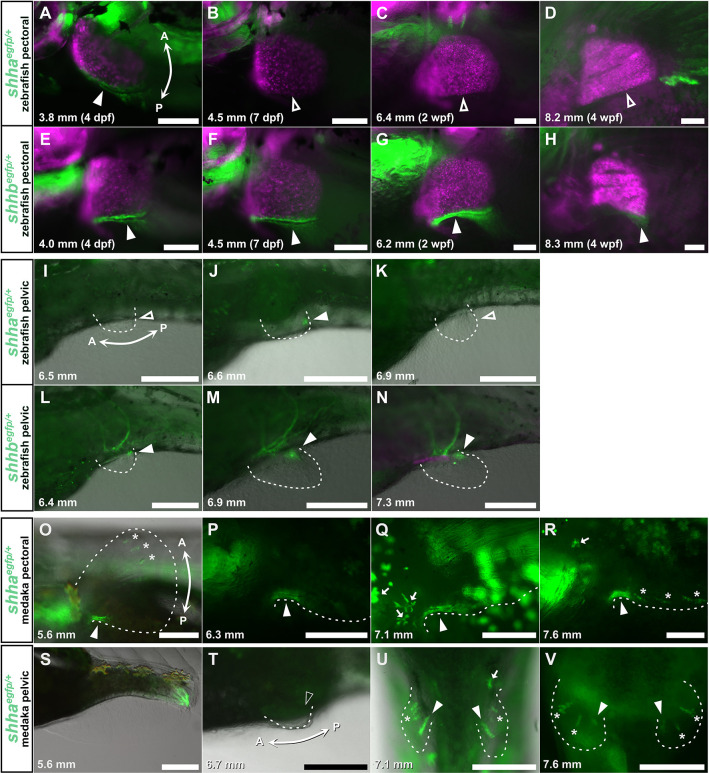
**Expression of *shha^egfp^* and *shhb^egfp^* in zebrafish, as well as *shha^egfp^* in medaka, during paired fin development.** (A-H) EGFP expression in pectoral fin development of *shha^egfp^* (A-D) and *shhb^egfp^* (E-H) zebrafish. (I-N) EGFP expression in pelvic fin development of *shha^egfp^* (I-K) and *shhb^egfp^* (L-N) zebrafish. (O-R) EGFP expression in pectoral fin development of *shha^egfp^* medaka. (S-V) EGFP expression in pelvic fin development of *shha^egfp^* medaka. All observations were conducted on ten or more larvae at each developmental stage. Magenta fluorescence indicates paired fin skeleton marked by *sox10:DsRed*. Filled white arrowheads denote EGFP expression and unfilled white arrowheads denote no EGFP expression. Dashed lines outline paired fin buds. Asterisks highlight EGFP expression at the tips of fin rays. White arrows indicate autofluorescence by leucophores. Double arrows mark the anterior (A)-posterior (P) axis. The standard length of individuals (in mm) is shown in the bottom left of each panel. wpf, weeks post-fertilization. Scale bars: 100 µm (A-H); 250 µm (I-V).

Given the development process of pectoral fins, the region-specific enlargement of the endochondral disk along the AP axis in *hhip^−/−^* zebrafish is regulated by the AP regionality derived from Hedgehog signaling. Hedgehog signaling in paired appendages is repressed by antagonization of Hhip (Chuang and McMahon, 1999; Koudijs et al., 2005). In paired appendages of vertebrates, Shh is expressed as Hedgehog ligands antagonized by Hhip. However, the expression patterns of Shh genes in the late development of paired fins when the endochondral disk grows and subdivides, remain unclear. Although zebrafish have two Shh genes, *shha* and *shhb* (Fig. S5), their expression has not been reported after 7 dpf. To investigate whether *shha* and *shhb* are expressed in the late development of the paired fins of zebrafish, we visualized them using a CRISPR-Cas9 knock-in approach (Auer et al., 2014; Kayo et al., 2023; Ota et al., 2016). We introduced an EGFP reporter in *shha* and *shhb* genes (*shha^egfp^*, *shhb^egfp^*; Fig. S6) and examined *shha* and *shhb* expression patterns in zebrafish (Fig. 3A-N). In the development of zebrafish pectoral fins, both *shha^egfp^* and *shhb^egfp^* were expressed in the posterior margin (Fig. 3A-H). However, *shha^egfp^* expression was observed until 4 dpf (Fig. 3A) and disappeared by 7 dpf (Fig. 3B-D), while *shhb^egfp^* expression continued beyond 4 dpf (Fig. 3E-H). This indicates that *shhb* is expressed for a longer period than *shha* until the subdivisions of the pectoral fin. In pelvic fin development, *shha^egfp^* expression in the posterior margin was short-lived (Fig. 3I-K), while *shhb^egfp^* expression in the posterior margin persisted until the initiation of pelvic girdle formation (Fig. 3L-N). Therefore, *shhb* is predominantly expressed during the late stages of pelvic fin development. In median fin development, neither *shha^egfp^* nor *shhb^egfp^* could be detected in the median fin primordia (Fig. S7).

While basal teleosts, including zebrafish, have both *shha* and *shhb* genes, teleosts in Acanthomorpha, including Japanese medaka, lost *shhb* and retained only *shha* (Fig. S5). If the *shha* expression pattern in Japanese medaka is similar to that in zebrafish, teleosts in Acanthomorpha might have lost late developmental Shh gene expression corresponding to that of zebrafish after 7 dpf in paired fins. However, in most teleosts in Acanthomorpha, four radials are conserved in the pectoral fin skeleton, similar to basal teleosts, which have both *shha* and *shhb* remaining (Tanaka et al., 2022). To explore how *shha* is expressed in Acanthomorpha, we generated a knock-in medaka line (*shha^egfp^*; Fig. 3O-V; Fig. S8A). The *shha^egfp^* medaka showed EGFP fluorescence in the posterior margin of the pectoral fin (Fig. 3O-R). In the pectoral fin development of Japanese medaka, *shha^egfp^* was expressed not only in the early stage (Fig. 3O,P) but also in the late stage when proximal radials begin to form (Fig. 3Q,R). In pelvic fin development, *shha^egfp^* was not expressed during the early budding (Fig. 3S,T) but was expressed from the mid-stage to the late stage (Fig. 3U,V). Similar to *shha^egfp^* and *shhb^egfp^* zebrafish, *shha^egfp^* medaka also showed no fluorescence in the median fin primordia (Fig. S8B,C). Therefore, the *shha* expression pattern in Japanese medaka was not equivalent to that of *shha* in zebrafish, but rather to that of *shhb* in zebrafish. These results suggest that teleosts largely retain Shh function until the late development of paired fins.

### The loss of *shhb* expression induces defects in paired fin skeletons

**Fig. 4.**
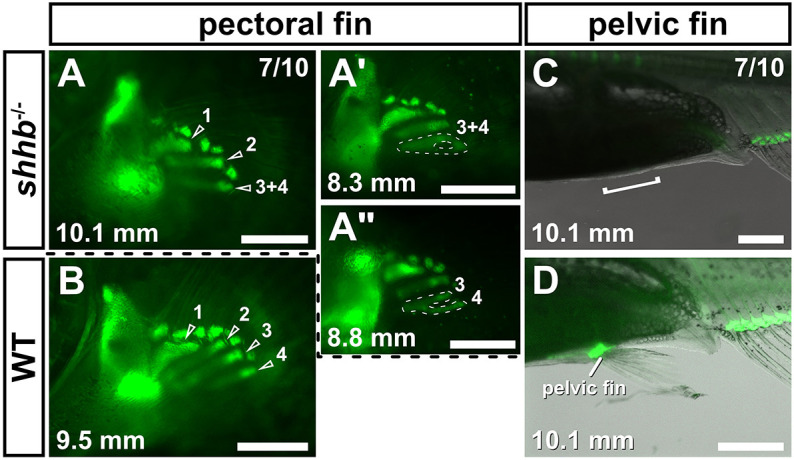
**Paired fin skeletons in *shhb****^−/−^* **zebrafish.** (A,B) Pectoral fin development in *shhb^−/−^* (A; *n*=7/10) and WT (B; *n*=10/10) zebrafish. For *shhb^−/−^* zebrafish, 7/10 larvae exhibited complete fusion of the third and fourth proximal radials in either the left or right pectoral fins. Some *shhb^−/−^* zebrafish exhibited a mild defect in the subdivision between the third and fourth proximal radials (A′,A″). Numbers indicate first to fourth proximal radials. (C,D) Pelvic fin development in *shhb^−/−^* (C; *n*=7/10) and WT (D; *n*=10/10) zebrafish. For *shhb^−/−^* zebrafish, 7/10 larvae exhibited pelvic fin loss, with 6/10 larvae exhibiting complete loss of both left and right pelvic fins, and 1/10 larvae exhibiting complete loss of only the left pelvic fin. The white bracket indicates the pre-anal region where the pelvic fins are formed in WT zebrafish. Green fluorescence indicates the endochondral skeleton marked by *col2a1a:EGFP*. The standard length of individuals (in mm) is shown in the bottom left of each panel. Scale bars: 250 μm (A-A″,B); 500 μm (C,D).

The knock-in analysis of Shh genes in zebrafish showed that the expression of *shhb* lasts longer than that of *shha*. There is a possibility that *shhb* expression primarily contributes to the growth of the endochondral disk and the number of radials in the pectoral fin skeleton. To investigate the functions of late Hedgehog signaling, the function of *shhb* in zebrafish was studied (Fig. 4). Using CRISPR-Cas9 genome editing in zebrafish, we introduced mutations in *shhb* and identified one *shhb* mutant that produced a non-functional truncated protein (Fig. S9).

In almost all *shhb^−/−^* zebrafish, three proximal radials were present in the pectoral fins (Fig. 4A). Some *shhb^−/−^* zebrafish showed an incomplete subdivision of the posterior endochondral part of the pectoral fin, where most posterior radials, the third and fourth proximal radials, were fused (Fig. 4A′,A″). Although the *shhb^−/−^* phenotype was mild compared to the severe phenotype of the *shha* complete loss mutants (Neumann et al., 1999), these data suggest that late Hedgehog signaling by *shhb* also contributes to the number of radials in the pectoral fin skeleton. Interestingly, in almost all *shhb* mutants, the entire pelvic fin skeleton, including the pelvic girdle, was absent (Fig. 4C). A similar loss of the pelvic fin skeleton has been reported in Japanese medaka without the *shha* fin enhancer (Letelier et al., 2021). This indicates that the *shhb* mutation in zebrafish leads to the loss of Hedgehog signaling, resulting in the absence of pelvic fin bud initiation. In zebrafish pelvic fin development, *shha* was rarely expressed while *shhb* expression dominated (Fig. 3I-N). These results suggest that *shhb* constitutes the entirety of Shh gene expression in the pelvic fin of zebrafish, and *shha* cannot compensate for the absence of *shhb*. Therefore, it was considered that the expression of *shhb*, rather than *shha*, regulates the development of the pelvic fin. Taken together, we conclude that *shhb* in zebrafish contributes to pectoral fin development along with *shha* and dominates pelvic fin development independently.

### Early expression of *hhip* is a characteristic of the pectoral fins of zebrafish

**Fig. 5.**
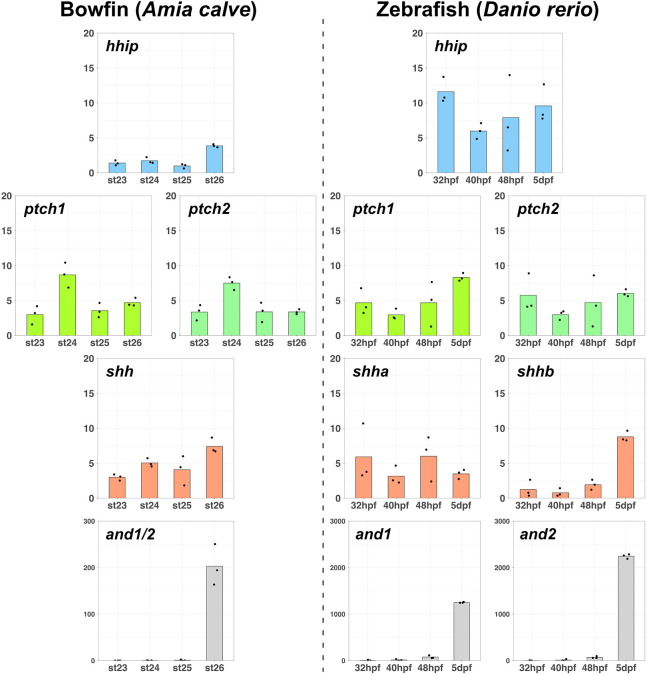
**Expression patterns of *hhip*-related genes in pectoral fins.** Bar graphs displaying expression levels (transcripts per million, TPM) at each developmental stage. The TPM of *hhip*, *ptch*, *shh* and *and* are in order from the top row. The *and* TPM distinguishes when fin fold formation began [bowfin, from stage (st) 26; zebrafish, from 5 dpf]. Each dot on the graphs represents the value of each replicate. The RNA-seq data of pectoral fins were obtained from previously published bowfin (Thompson et al., 2021) and zebrafish (Kudoh et al., 2024) studies.

The *hhip* and *shh* analyses indicated that *hhip* expression during early development, when both duplicated Shh genes were expressed, regulated pectoral fin elaboration in teleosts. However, it is unclear whether early *hhip* expression is characteristic of teleosts. To compare *hhip* expression patterns in the pectoral fins of zebrafish with those of basal species, we used previously published RNA-sequencing (RNA-seq) data of pectoral fins in the brownbanded bamboo shark (Onimaru et al., 2021), bowfin (Thompson et al., 2021), and zebrafish (Kudoh et al., 2024). For this analysis, we re-analyzed the RNA-seq data using the same pipeline (Patro et al., 2017). We selected *hhip*-related genes (*hhip*, *ptch1*, *ptch2*, and *shh*), and *actinodin* (*and*) genes for comparison of the developmental process (Fig. 5; Fig. S10). *ptch1* and *ptch2* encode Hedgehog signaling receptors that compete with Hhip. *and* encodes a structural protein specific to fin folds, a structure covering the outer margin of the endochondral disk and forming fin rays (Zhang et al., 2010). We used *and* genes to distinguish the stages before and after the beginning of fin fold formation in each species (Fig. 5; Fig. S10, bottom row). The expression level (transcripts per million, TPM) of *hhip* in zebrafish was higher than that of bowfin, implying that *hhip* expression increases from basal actinopterygians to teleosts (Fig. 5). Additionally, for inter-species comparison of *hhip* expression, we scaled the *hhip* TPM by the sum of *ptch1* and *ptch2* TPM (Fig. S10, top row). After fin fold formation, the scaled *hhip* values for each species were almost the same. Before fin fold formation, however, the scaled value of zebrafish was higher than after fin fold formation, while those of the brownbanded bamboo shark and bowfin were lower than after fin fold formation. Taken together, the significant expression of *hhip* before fin fold formation was characteristic of pectoral fin development in zebrafish.

### The number of radials in the median region modulates the width of pectoral fins in fishes along the AP axis

We then examined whether the pectoral fin skeleton of *hhip^−/−^* zebrafish is comparable to those of basal species. Morphologically, the numerous radials in the pectoral fin of basal species are derived from the posterior region because they branch off from the metapterygium, the posterior tribasal bone (Gegenbaur, 1865; Hawkins et al., 2021; Mabee, 2000; Onimaru et al., 2015; Tanaka, 2018). As a result, teleosts have been considered to have lost the posterior region and radials, leading to pectoral fin elaboration. Here, we identified a genetic AP regionality in teleosts and examined the region involved in pectoral fin elaboration.

**Fig. 6.**
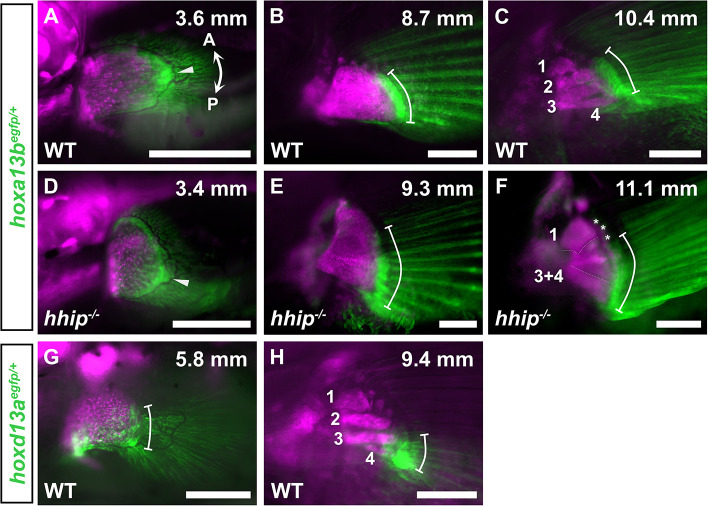
**Expression of EGFP by *hoxa13b^egfp^* and *hoxd13a^egfp^* in pectoral fin development of zebrafish.** (A-F) EGFP expression induced by *hoxa13b^egfp^* in pectoral fin development of WT (A-C) and *hhip^−/−^* (D-F) zebrafish. (G,H) EGFP expression induced by *hoxd13a^egfp^* in pectoral fin development of WT. Numbers indicate the first to fourth proximal radials. White arrowheads indicate significant expression of EGFP by *hoxa13b^egfp^*. White bars represent the range of EGFP expression. White asterisks denote EGFP expression by *col2a1a:egfp* on the distal radials. Observations were conducted on more than five larvae at each developmental stage. Magenta fluorescence indicates the paired fin skeleton marked by *sox10:DsRed*. Double arrows indicate the anterior (A)-posterior (P) axis. The standard length of individuals (in mm) is shown in the top right of each panel. Scale bars: 250 µm.

To identify the AP axis genetically, we visualized the expression patterns of the *hoxa13* and *hoxd13* genes in zebrafish. These genes are expressed along the AP axis in some chondrichthyans, basal actinopterygians, and teleosts (Ahn and Ho, 2008; Davis et al., 2007; Freitas et al., 2007; Sordino et al., 1995; Tulenko et al., 2016). We generated CRISPR-Cas9 knock-in zebrafish lines (*hoxa13b^egfp^*, *hoxd13a^egfp^*; Fig. S11). Prior to the subdivision of the endochondral disk in the pectoral fin, *hoxa13b^egfp^* was initially expressed in the distal margin (Fig. 6A), and later its expression was limited to the posterior region of the endochondral disk (Fig. 6B). Similarly, *hoxd13a^egfp^* was expressed in the posterior region of the endochondral disk (Fig. 6G). As the pectoral fin subdivided, *hoxa13b^egfp^* was expressed on the distal margin covering the second, third, and fourth proximal radials (Fig. 6C), while *hoxd13a^egfp^* was expressed on the distal margin covering the third and fourth proximal radials (Fig. 6H). In *hhip^−/−^* zebrafish, *hoxa13b^egfp^* was expressed in the distal margin, but EGFP was highly expressed in posterior cells compared to WT zebrafish (Fig. 6A,D, arrowheads). Throughout the subdivision, *hoxa13b^egfp^* was expressed not only on the distal margin of the most posterior radials equivalent to the third and fourth radials in WT zebrafish but also on the distal margin of the increased radials equivalent to the second radial in WT zebrafish (Fig. 6E,F). These results suggest that the pectoral fin of zebrafish is regionalized by distinct patterns of *hoxa13* and *hoxd13* expression, and that the number of radials in *hhip* mutants increases in the median region where *hoxa13b* is expressed.

During development of the pelvic fins in zebrafish, *hoxa13b^egfp^* was expressed throughout the entire distal region (Fig. S12A-C), while *hoxd13a^egfp^* was expressed specifically in the posterior region where the PoR was formed (Fig. S12D,E). In the caudal fin primordium, *hoxa13b^egfp^* was evenly expressed until 2 dpf (Fig. S12F), but then the median expression, including the second and third hypurals, was lost (Fig. S12G,H), whereas *hoxd13a^egfp^* was only expressed in blood vessels and not in the caudal fin primordium (Fig. S12I-K). This suggests that both the pelvic and caudal fins of zebrafish exhibit regionality based on *hoxa13* or *hoxd13* expression.

## DISCUSSION

**Fig. 7.**
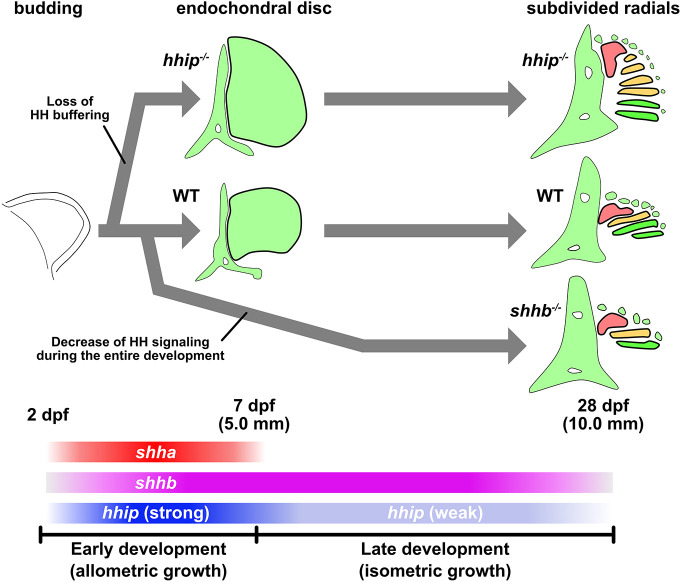
**Summary of pectoral radial changes in zebrafish.** Diagram illustrating the pectoral fin skeleton at each developmental stage. The length under the dpf stage indicates approximate standard length. The color bars with gene names indicate gene expression periods. Colors in the 28 dpf section indicate the anterior region where neither *Hoxa13* nor *Hoxd13* is expressed (red), the median region where *Hoxa13* is expressed but *Hoxd13* is not expressed (orange), and the posterior region where both *Hoxa13* and *Hoxd13* are expressed (green).

Our genetic and developmental analyses in zebrafish demonstrated the detailed growth pattern of the endochondral disk of the pectoral fin along the AP axis. In *hhip* mutants, the endochondral disk expanded along the AP axis, and the number of radials increased (Fig. 1C; Fig. S1F). *hhip* was expressed in the entire pectoral fin bud during early development, but only in the posterior margin during late development (Fig. S4A-E; Koudijs et al., 2005). This pattern was associated with the early excessive growth of the endochondral disk in *hhip* mutants until approximately 7 dpf (standard length=5.0 mm; Fig. S1B-E). In contrast, *shhb* mutation induced a decrease in Hedgehog signaling during both early and late development, resulting in a defect in pectoral fin radials (Fig. 4A). From these results, we suggest that the endochondral disk has two growth phases: allometric growth during early development, and isometric growth during late development (Fig. 7). During early development, the ratio of the AP axis to the PD axis (AP-PD ratio) in the endochondral disk can change, resulting in allometric growth (Fig. 7). Therefore, the number of pectoral fin radials is largely determined by the early growth of the endochondral disk. Conversely, late growth of the endochondral disk may result in an isometric AP-PD ratio. Although the number of radials is largely dependent on the early allometric growth of the endochondral disk, the Hedgehog signaling-promotive growth is buffered by *hhip* negative feedback. Both *shha* and *shhb* were expressed in the pectoral fins of zebrafish, while only *shha* was expressed in Japanese medaka (Fig. 3). Nevertheless, both fishes have the same number of radials in their pectoral fin skeletons. Although other differences, such as the activities of receptors or regulatory enhancers, may also regulate the strength of Hedgehog signaling in detail (Letelier et al., 2018; Lettice et al., 2017; Lopez-Rios et al., 2014), we suggest that the negative feedback effect of *hhip* buffers the different strengths of Hedgehog signaling to a certain level, generating four radials across teleosts. The inter-species analysis between zebrafish and basal species showed that the early *hhip* expression is characteristic of zebrafish (Fig. S10). Therefore, the varying number of pectoral fin radials in basal species may reflect non-buffered Hedgehog signaling activity. In summary, the data suggest that pectoral fin elaboration in teleosts depends on *hhip*-mediated negative feedback of Hedgehog signaling during early development.

The phenotypes in *hhip^−/−^* zebrafish support a potential developmental mechanism in the evolution of paired fins from the ancestral form to the teleost-specific derived form. First, based on observation of the developmental process (Fig. 2A-F), the increase in radials in the pectoral fins of *hhip^−/−^* zebrafish occurred in the median region occupied by the second proximal radial, rather than in the posterior region. During the development of pectoral fins in chondrichthyans and basal actinopterygians, the anterior (Fig. S13A, red) and posterior basal radials (Fig. S13A, green) are formed first, followed by the other median radials (Fig. S13A, orange), which fill in the gap between the anterior and posterior radials (Davis et al., 2004; Dillman and Hilton, 2015; Mabee and Noordsy, 2004; Onimaru et al., 2016). The subdivision order of the endochondral disk is similar between basal species and *hhip^−/−^* zebrafish, with the radials in the median region being the last to form (Fig. 2C). Second, Hox gene expression supports the AP regionality based on endochondral subdivisions during pectoral fin development in *hhip^−/−^* zebrafish (Fig. 6). *Hoxa13* is expressed in the distal margin of the median and posterior radials during late development of paddlefish (Davis et al., 2007). *Hoxd13* is expressed in the posterior region of the pectoral fins in catshark and paddlefish (Davis et al., 2007; Freitas et al., 2007; Tulenko et al., 2016). These Hox expression patterns correspond to those in zebrafish (Fig. 6; Fig. S12A-E, Fig. S13A). Overall, the expression of *Hoxa13* and *Hoxd13* determines the anterior (Fig. 7, red; neither *Hoxa13* nor *Hoxd13* are expressed), median (Fig. 7, orange; *Hoxa13* is expressed but *Hoxd13* is not expressed), and posterior (Fig. 7, green; both *Hoxa13* and *Hoxd13* are expressed) regions in the pectoral fin skeleton. EGFP expression induced by *hoxa13b^egfp^* in *hhip^−/−^* zebrafish showed anterior expansion, eventually reaching the posterior border of the first proximal radials, supporting the above regional distinctions (Fig. 6F). Lastly, our expression and mutation analyses highlighted the importance of the median region for pectoral fin elaboration. *ptch1* was expressed only in the posterior margin in WT (Fig. S4F), while *ptch1* expression reached both the median and posterior regions in *hhip^−/−^* zebrafish (Fig. S4G). Based on these expression patterns, Hedgehog signaling affected only the posterior region in *shhb^−/−^* zebrafish, which exhibited fusion of posterior radials, and affected both the median and posterior regions in *hhip^−/−^* zebrafish, which had excessive radials in the median region. We suggest that the median region is susceptible to Hedgehog signaling, which can increase the number of radials response to the signaling. By contrast, the posterior region cannot increase radials but requires a certain degree of Hedgehog signaling to generate normal third and fourth radials. Although it has been believed that the pectoral fins of teleosts lost posterior radials branched from the metapterygium in the ancestral form and decreased to only four radials, we suggest that pectoral fin elaboration along the AP axis occurs through the reduction of radials in the median Hedgehog-susceptible region (Fig. 7, orange). In addition, pelvic fins also exhibited an increase in radials in *hhip^−/−^* zebrafish (Fig. 2I; Fig. S13B). The pelvic fin skeletons of chondrichthyans, sturgeon, and paddlefish possess multiple radials, while there are fewer radials in the pelvic fin skeletons of polypterus, bowfin, and teleosts (Grandel and Schulte-Merker, 1998; Hilton, 2011; Jarvik, 1980; Mabee and Noordsy, 2004; Molnar et al., 2017). Therefore, Hedgehog signaling may be involved in variation of the number of paired fin radials.

These patterns can be extended to the morphogenesis of the caudal fin in teleosts. In the caudal fin skeleton of teleosts, the number of hypurals is decreased in parallel with the elaboration of paired fins (Desvignes et al., 2018). In *hhip^−/−^* zebrafish, hypural 2 was branched and three hypurals were formed (Fig. 2M), suggesting that caudal fin morphogenesis is regulated by axis formation similar to the AP regulation for paired fins. *alx4*, an anterior-specific gene in the developing paired fins, is expressed at both the dorsal and ventral margins during caudal fin development, suggesting that the caudal fin possesses bipolar anterior regulation (Desvignes et al., 2022). Additionally, *gdf5* mutation in zebrafish, which affects the formation of posterior radials in pectoral fins, disrupts the formation of the dorsal hypurals 3-5, suggesting that the dorsal region of the caudal fin may correspond to the posterior region of pectoral fins (Waldmann et al., 2021). These reports on molecular markers suggest that the dorsal-ventral axis in the caudal fin is regulated by a mechanism corresponding to the AP axis in paired fins (Fig. S13B). Previous studies have identified various phenotypes in the caudal fin skeleton (Abe et al., 2014; Moriyama et al., 2012), but our results are the first to report Hedgehog-susceptible and region-specific phenotypes. Although neither *shha* nor *shhb* expression was detected in the endochondral primordium of the caudal fin skeleton (Fig. S7A,B,E,F), Shh ligands secreted by other organizers such as the notochord may regulate hypural development (Hadzhiev et al., 2007). We suggest that Hedgehog susceptibility in the caudal fin is involved in the morphological evolution of the caudal fin skeleton. The number of hypurals decreased in the morphological change from the heterocercal form of chondrichthyans and basal actinopterygians to the homocercal form of teleosts (Desvignes et al., 2018), and this morphological change occurred approximately concurrently with paired fin elaboration. Therefore, repression of Hedgehog signaling might affect not only paired fin evolution but also caudal fin evolution from the ancestral form to the teleost form. However, the caudal fin skeletons in basal species have more hypurals than those in *hhip^−/−^* zebrafish (Cumplido et al., 2024). An increase in the number of ural vertebrae itself will be required to replicate the complete pattern of the caudal fin skeleton in basal species. Additional mutations modulating axial elongation (Aires et al., 2019; Cumplido et al., 2024; Kingsley et al., 2024) may generate sufficient ural vertebrae and hypurals to obtain a caudal fin skeleton of teleosts that is closer to that of basal species.

In *hhip^−/−^* zebrafish, the dorsal fin radials increased, but the anal fin skeleton was completely absent (Fig. 1G-K). The opposite phenotypes in the median fin skeletons seem inexplicable, but the function of *hhip* on the dorsal-ventral regionalization in the somites may resolve the inconsistency. *hhip* is expressed in the horizontal boundary cells of somites in teleosts and regulates the dorsal-ventral boundary of somites (Fig. S4A; Abe et al., 2019). Mutation of *hhip* in Japanese medaka expands the expression of *zic1*, a dorsal marker of somites, largely into the ventral part of somites (Abe et al., 2019). Since the somite cells contribute to the median fins as skeletal components, the median fin phenotypes presented here may be induced by the dorsalized somites, giving rise to an increase in dorsal fin components and a loss of the ventral ones that differentiate into anal fin skeletons. It is possible that regulation within somites might be as important as that within fin buds for median fin development, although the effect of Shh within median fin buds has been the focus of studies so far (Dahn et al., 2007; Höch et al., 2021; Letelier et al., 2018, 2021).

Shh regulation in paired fin development differs in several aspects between basal teleosts (such as zebrafish) and Acanthomorpha teleosts (such as medaka). The first difference lies in the mechanism underlying the repression of Hedgehog signaling. Our results showed that *hhip* mutant zebrafish exhibited an increase in radials, while *gli3* mutant zebrafish exhibited no abnormalities in the fin skeletons (Fig. S2). In contrast, a previous study demonstrated that *gli3* mutant medaka exhibited an increase in radials in the paired and dorsal fin skeletons (Letelier et al., 2021). We hypothesized that the genes involved in the repression mechanism of Hedgehog signaling may vary while still maintaining the number of radials. Knockout of *hhip* in Japanese medaka will help elucidate the generalizability and variability in Hedgehog repression that regulates pectoral fin development. The second difference lies in how Shh gene expression is regulated between zebrafish and Japanese medaka (Fig. 3). In zebrafish, both *shha* and *shhb* regulate paired fin development (Fig. 3A-N); however, only *shha* regulates development in medaka (Fig. 3O-V). The expression of Shh genes at the posterior margin of paired fins is regulated by a single cis-regulatory element called the ZPA regulatory sequence (ZRS) (Letelier et al., 2018; Lettice et al., 2003; Sagai et al., 2005). The ZRS is located in intron 5 of *lmbr1*, a gene located on the same chromosome as Shh genes, and is common among gnathostome genomes. In basal teleosts, the *lmbr1* paralog has been conserved in the genomic region upstream of *shha* (Fig. S5). Conversely, the genomic region upstream of *shhb* has lost the *lmbr1* paralog and some neighboring genes, leading to a gene desert. Considering that *shhb* in the paired fins of zebrafish was expressed for a similar duration as *shha* in medaka, it is possible that the genomic region upstream of *shhb* in zebrafish contains a conserved paired fin enhancer equivalent to the ZRS located within the *lmbr1* intron. However, the expression of *shha* in the paired fins of zebrafish has likely been modulated to be shorter than that of *shhb* in zebrafish and *shha* in Japanese medaka. In summary, the regulation system of Hedgehog signaling during fin development varies even between zebrafish and Japanese medaka. In this study, although we suggest that fin elaboration in zebrafish is regulated by negative feedback of *hhip*, the developmental regulation of Hedgehog signaling appears to have diversified among teleosts. Further genetic analyses in other teleosts will help uncover the precise evolutionary mechanism of fin skeleton development.

## MATERIALS AND METHODS

### Fish care and strains

The following transgenic zebrafish lines were used in this study: *col2a1a:EGFP* (Hamada et al., 2019) and *sox10:DsRed* (Yoshida et al., 2020). Zebrafish and medaka were housed at 28°C with a 14 h light cycle. The standard length of individuals was used as an indicator of individual body size instead of the date of development since zebrafish of the same age often have different body sizes (Parichy et al., 2009). The *hhip^−/−^* zebrafish had two different mutations (H215RfsX1, F242SfsX8). Homozygous alleles of both mutations (H215RfsX1/H215RfsX1, F242SfsX8/F242SfsX8) and compound heterozygous alleles (H215RfsX1/ F242SfsX8) induced the *hhip* mutant phenotypes. All *shhb^−/−^* zebrafish that we observed possessed homozygous alleles (G122LfsX1/G122LfsX1).

### Preparation of sgRNAs

All single guide RNAs (sgRNAs) were designed using CRISPRscan (Moreno-Mateos et al., 2015) and CHOPCHOP (Labun et al., 2019) CRISPR online tools. Template DNA for sgRNA synthesis was PCR-amplified using the crRNA/tracrRNA sequence primer AAAAGCACCGACTCGGTGCCACTTTTTCAAGTTGATAACGGACTAGCCTTATTTTAACTTGCTATTTCTAGCTCTAAAAC with the forward primer AAAAGCACCGACTCGGTGCC and the reverse primer TAATACGACTCACTATAggxxxxxxxxxxxxxxxxxxGTTTTAGAGCTAGAAATAGCA (for T7 polymerase). Lowercase letters indicate genome-targeting sequences (either 19 or 20 bases) in sgRNAs. The genome-targeting sequences in sgRNAs used in this study are shown in Table S1. After PCR amplification with KOD -plus- neo polymerase (Toyobo), PCR products were purified using a PCR purification kit (Cica). The obtained template DNA was used for *in vitro* transcription of sgRNAs using a CUGA^®^7 gRNA synthesis kit (Nippon Gene). sgRNAs were purified using the CUGA^®^7 gRNA synthesis kit (Nippon Gene).

### Microinjection for mutagenesis and knock-in

sgRNAs and Cas9 nuclease were co-injected into one-cell-stage zebrafish and medaka embryos. Each embryo received an injection of a solution containing 10 ng/μl of sgRNA for digesting genomic DNA and 250 ng/μl of Cas9 nuclease (Integrated DNA Technologies). For knock-in samples, we also added 10 ng/μl of sgRNA for digesting BaitD and 7.5 ng/μl of phenol-chloroform to extract purified plasmid. To minimize leaky EGFP expression, we used the pUC-BaitD-Xhbb-EGFP plasmid, known for stable EGFP expression in target tissues (Kayo et al., 2023). The injection volume was adjusted to result in the death of approximately 50% of injected embryos within 1 week after injection. These injection mixtures were introduced into one-cell-stage eggs, following established protocols for zebrafish (Kawakami et al., 2016) and medaka (Kinoshita, 2009).

### Genotyping and sequencing of mutant fish

The genotype of mutant fish was determined using a heteroduplex mobility assay (HMA) (Ansai et al., 2014). The sequences of mutant alleles isolated from F1 or later generations were determined through direct Sanger sequencing of the PCR products using the primer pairs shown in Table S2. All mutations present in these mutants are listed in Table S3.

### Genotyping and sequencing of knock-in fish

For knock-in insertion mapping, we collected fluorescent F1 fish at 2-4 dpf and extracted genomic DNA using standard protocols. The insertion status was examined from either the 5′ side or the 3′ side of the insertion. For example, when checking from the 5′ side of the insertion, a PCR reaction was performed using a 5′ primer specific to each target gene (upstream of the expected insertion site) and a 3′ primer specific to the donor plasmid, pUC-BaitD-Xhbb-EGFP. The primers used for insertion mapping in this study are shown in Table S2. The ‘knock-in check’ primers were designed on the 5′ side (fw) and the 3′ side (rv) of the donor plasmid, respectively. The sequences of mutant alleles isolated from F1 or later generations were determined by direct Sanger sequencing of the PCR products.

### Bone staining with Alizarin Red

Vital bone staining was performed with Alizarin Red S (AR) solution in water, which was prepared before use, as previously described with slight modifications (Yoshida et al., 2020). In this study, zebrafish were transferred to the AR solution and incubated for 2 h at 28.5°C. Following bone staining, the zebrafish were washed several times with system water and examined under a Leica M205 FA stereomicroscope. Images were taken using a Leica DFC 360 FX camera.

### Whole-mount *in situ* hybridization

Probe sequences for *in situ* hybridization were cloned into the pBluescript-II-SK(+) vector using PCR from zebrafish cDNA and the In-Fusion^®^ HD Cloning Kit (Takara Bio Inc.). PCR primers are listed in Table S4. The zebrafish *hhip* probe had been previously used in research (Koudijs et al., 2005). *In situ* hybridization was performed following the protocol outlined in a previous study (Woltering et al., 2009) with a slight modification of the number of washes increasing one or more times during every washing process.

### Phylogenetic analysis

To infer individual gene family trees, amino acid sequences were retrieved from aLeaves (Kuraku et al., 2013) and manually curated. Multiple sequence alignment was performed using MAFFT (v.7.525) (Katoh et al., 2002) with the option ‘-linsi’. The aligned sequence sets were then processed using trimAl (v.1.4.1) (Capella-Gutiérrez et al., 2009) with the option ‘-automated1’. Molecular phylogenetic trees were inferred using RAxML (v.8.2.12) (Stamatakis, 2014) with the ‘-m PROTGAMMAAUTO -f a -# 1000’ options.

### RNA-seq data analysis

The RNA-seq re-analyses of pectoral fins were performed using the publicly available RNA-seq data of the brownbanded bamboo shark (Onimaru et al., 2021), bowfin (Thompson et al., 2021), and zebrafish (Kudoh et al., 2024). The Salmon pipeline was used for transcriptome assembly and quantification (Patro et al., 2017). The transcript-level counts were then summarized to gene-level counts using tximport v.1.30.0 (Soneson et al., 2015). Reference transcriptome data for the brownbanded bamboo shark (https://github.com/Squalomix/sequences), bowfin (https://github.com/AndrewWT/AmiaGenomics), and zebrafish (Ensembl GRCz11 cDNA; https://ftp.ensembl.org/pub/release-111/fasta/danio_rerio/cdna/) were employed. To identify non-annotated genes in the brownbanded bamboo shark and bowfin, BLASTN v.2.15.0 was performed against these reference transcriptome data. For the brownbanded bamboo shark, coding gene sequences from the elephant shark (Callorhinchus_milii-6.1.3) were used, and for the bowfin, sequences from the spotted gar (LepOcu1) were used.

### Fluorescence imaging

Zebrafish and medaka were anesthetized by immersion in 0.025% MS222 and placed in 3% agarose gel/E3 on a glass slide. All fluorescence images were taken using a Leica M205 FA microscope system and photographed with a Leica DFC 369 FX camera.

### Statistical analysis

The number of skeletal components and the length of pectoral fin primordia were measured at specific time points during the developmental process using *col2a1a:EGFP* fluorescence and Leica Application Suite X (LAS X, Leica). Differences in the number of skeletal elements and the length between mutant and WT zebrafish were tested using an unpaired *t*-test in GraphPad Prism software.

## Supplementary Material



10.1242/develop.202526_sup1 Supplementary information
