# Supplementary material for: Anterior-posterior constraint on Hedgehog signaling by hhip in teleost fin elaboration
Source: Development. 2024 Nov 18;151(22):dev202526. doi: 10.1242/dev.202526 (PMC11607692; doi:10.1242/dev.202526)
Supplement: Supplementary information [file develop-151-202526-s1.pdf]

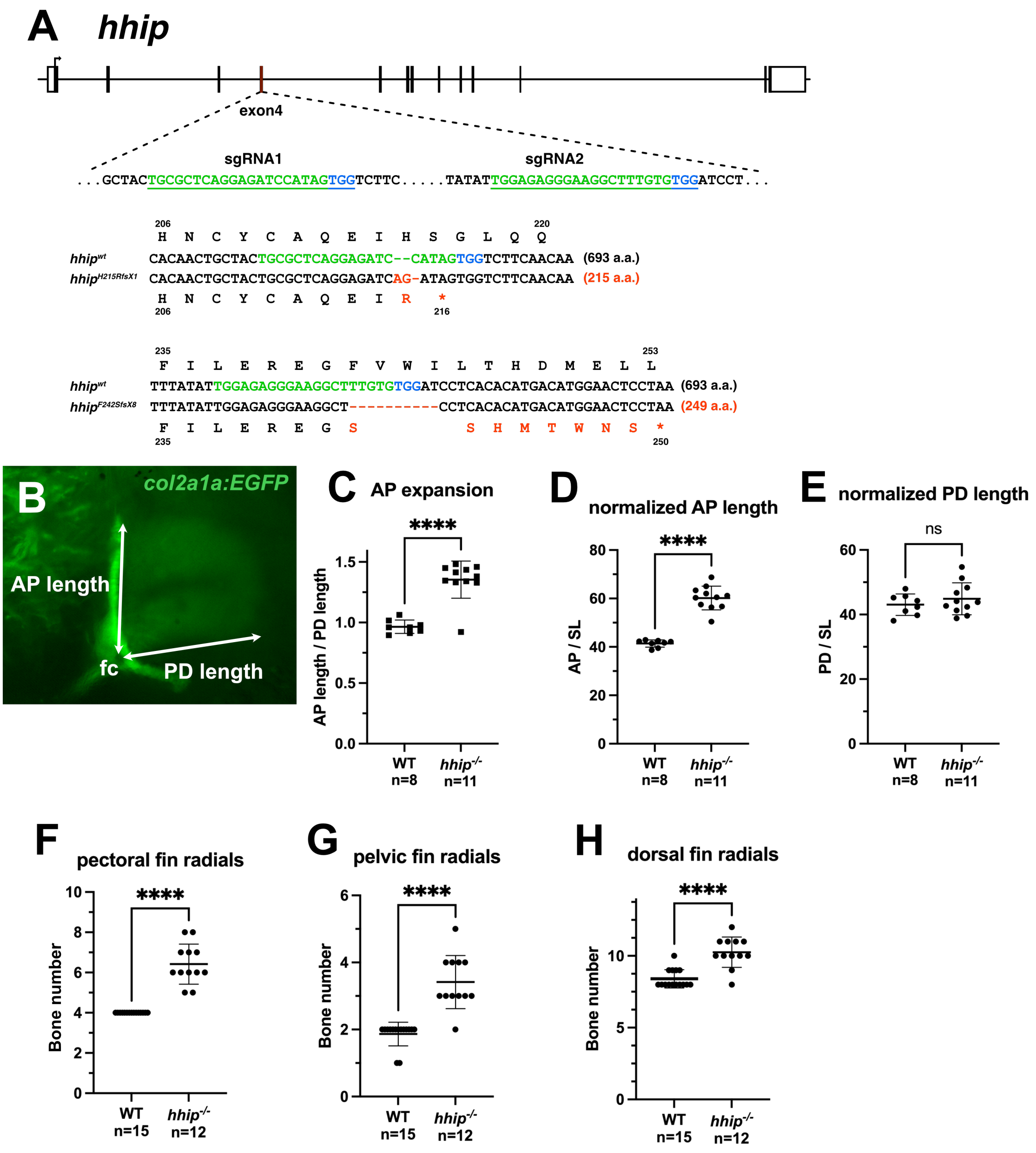


**Fig. S1. *hhip* mutation in zebrafish.**

(A) Sequence showing the *hhip* mutations. The sequences in blue indicate the protospacer adjacent motif (PAM), in green indicate the recognition sequence for the sgRNA target and in red indicate the mutation site. (B) Method for measuring the length of the pectoral fin skeleton. The anterior-posterior (AP) length was measured from the foramen coracoideus (fc) to the anterior margin of the endochondral disc. The proximal-distal (PD) length was measured from the foramen coracoideus to the distal margin of the endochondral disc. Green fluorescence indicates the endochondral skeleton marked by *col2a1a:EGFP*. (C-E) Quantification of the length of pectoral fins in *hhip* mutants. Fish

(SL=5.0–7.5 mm) that had not initiated the subdivision of the endochondral disc were used in these analyses. An unpaired *t*-test was used for the statistical analysis of pectoral fin length. (C) AP length normalized by PD length. Pectoral fins of *hhip*$^{-/-}$ zebrafish expanded along the AP axis. ****$p$<0.0001. WT: mean, 0.9651; *hhip*$^{-/-}$: mean, 1.354. (D, E) AP and PD lengths normalized by standard length (SL). The pectoral fins of *hhip*$^{-/-}$ zebrafish did not expand along the PD axis. (D) ****$p$<0.0001. WT: mean, 41.41; *hhip*$^{-/-}$: mean, 60.18. (E) $p$=0.3077. WT: mean, 43.06; *hhip*$^{-/-}$: mean, 44.88. (F-H) Quantification of the number of radials in pectoral (F), pelvic (G), and dorsal fins (H). The number of pelvic fin radials did not include the PoR. Fish (SL>8.0 mm) with complete formation of skeletal elements were used in these analyses. An unpaired *t*-test was used for the statistical analysis of skeletal element number. (F) ****$p$<0.0001. WT: mean, 4.00; *hhip*$^{-/-}$: mean, 6.417. (G) ****$p$<0.0001. WT: mean, 1.867; *hhip*$^{-/-}$: mean, 3.417. (H) ****$p$<0.0001. WT: mean, 8.40; *hhip*$^{-/-}$: mean, 10.25.

Development • Supplementary information

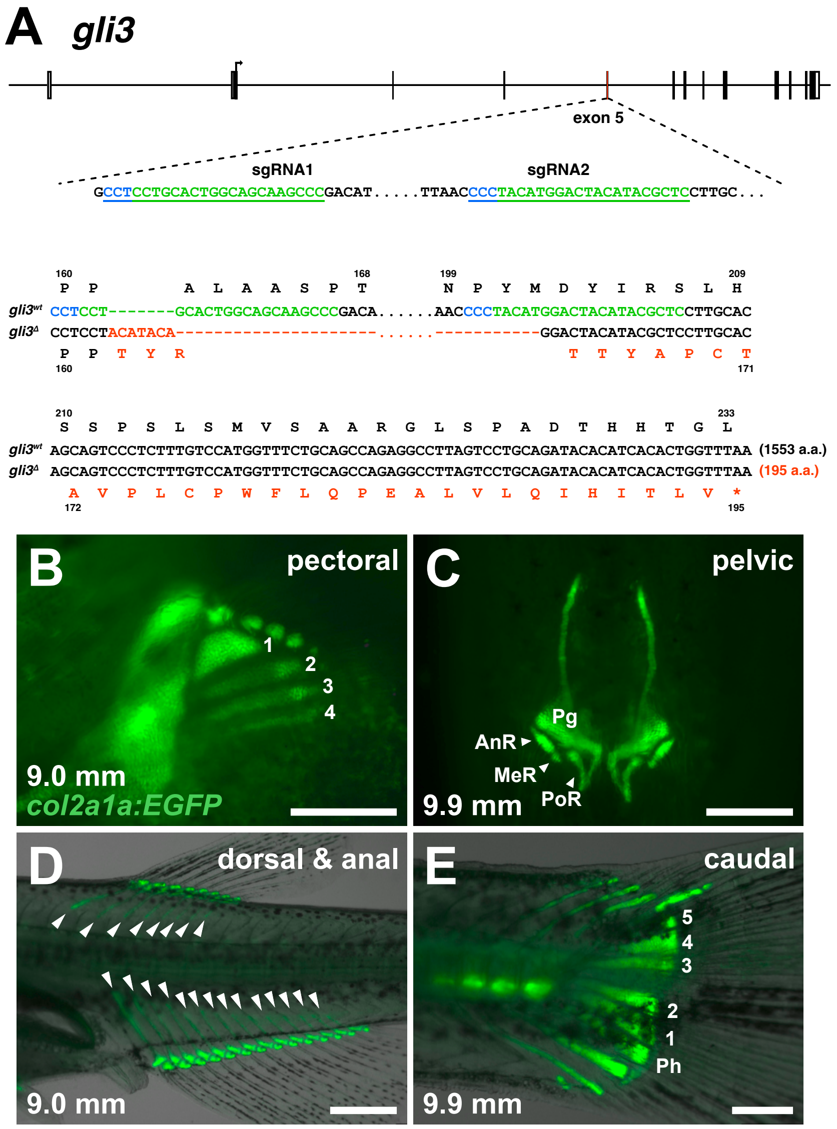


**Fig. S2. *gli3* mutation in zebrafish.**

(A) Sequence showing the *gli3* mutations. The sequences in blue indicate the protospacer adjacent motif (PAM), in green indicate the recognition sequence for the sgRNA target, and in red indicate the mutation site. (B) Pectoral fin skeleton in $gli3^{\Delta/\Delta}$. Numbers indicate the 1st to 4th proximal radials. (C) Pelvic fin skeleton in $gli3^{\Delta/\Delta}$. AnR, anterior large radial; MeR, medial small radial; Pg, pelvic girdle; PoR, posterior elongated radial. (D) Dorsal and anal fin skeletons in $gli3^{\Delta/\Delta}$. Closed white arrowheads indicate the radial bones. (E) Caudal fin skeleton in $gli3^{\Delta/\Delta}$. Numbers indicate the 1st to 5th hypurals. Ph, parhypural. Observations were performed on five or more larvae of $gli3^{\Delta/\Delta}$ zebrafish. Green fluorescence indicates the endochondral skeleton marked by *col2a1a:EGFP*. The standard length of individuals (in mm) is shown in the bottom left in panels (B-E). Scale bars: 250 µm (B, C, E); 500 µm (D).

Development • Supplementary information

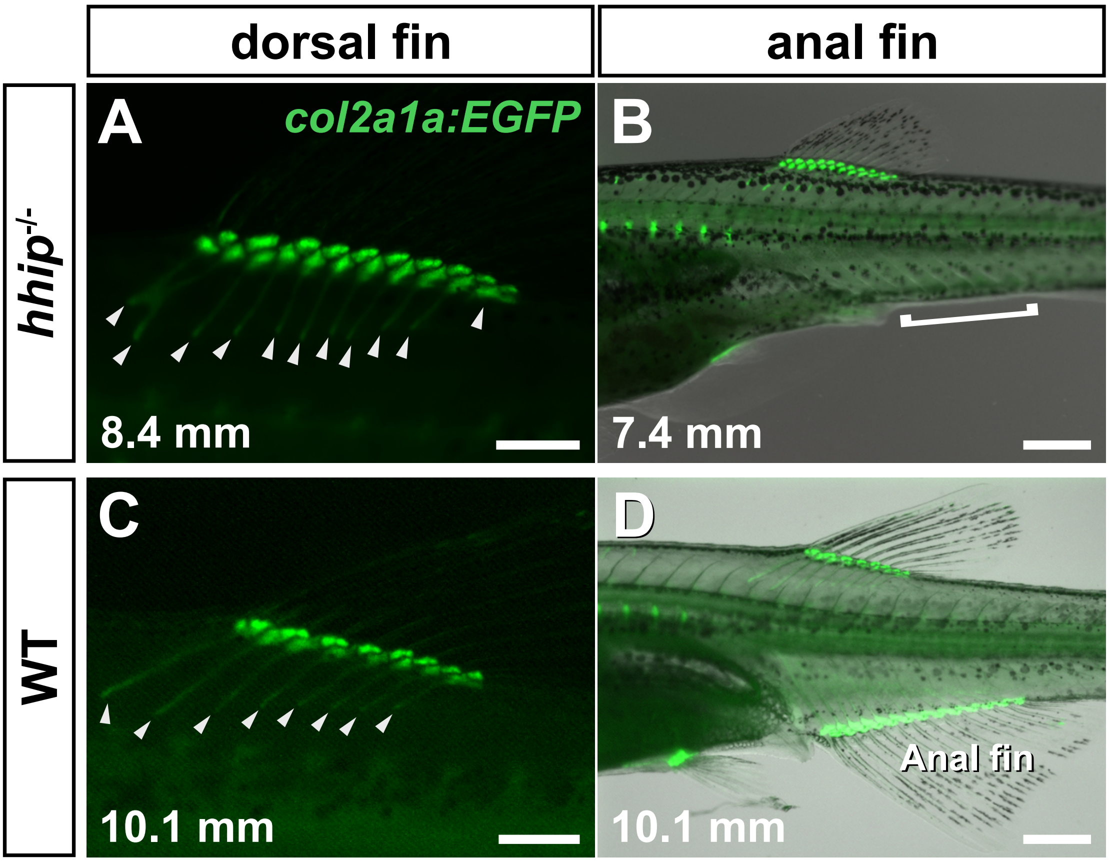


**Fig. S3. Dorsal and anal fin skeletons in *hhip* mutant zebrafish.**

(A, B) Dorsal and anal fin skeletons in *hhip*$^{-/-}$ zebrafish. (C, D) Dorsal and anal fin skeletons in WT zebrafish (D). White arrowheads indicate the radial bones. The white bracket highlights the post-anal region where the anal fin is formed in WT zebrafish. These observations were made on 10 or more WT and *hhip*$^{-/-}$ larvae. Green fluorescence indicates the endochondral skeleton marked by *col2a1a:EGFP*. The standard length of individuals (in mm) is shown in the bottom left of each panel. Scale bars: 250 µm (A, C); 500 µm (B, D).

Development • Supplementary information

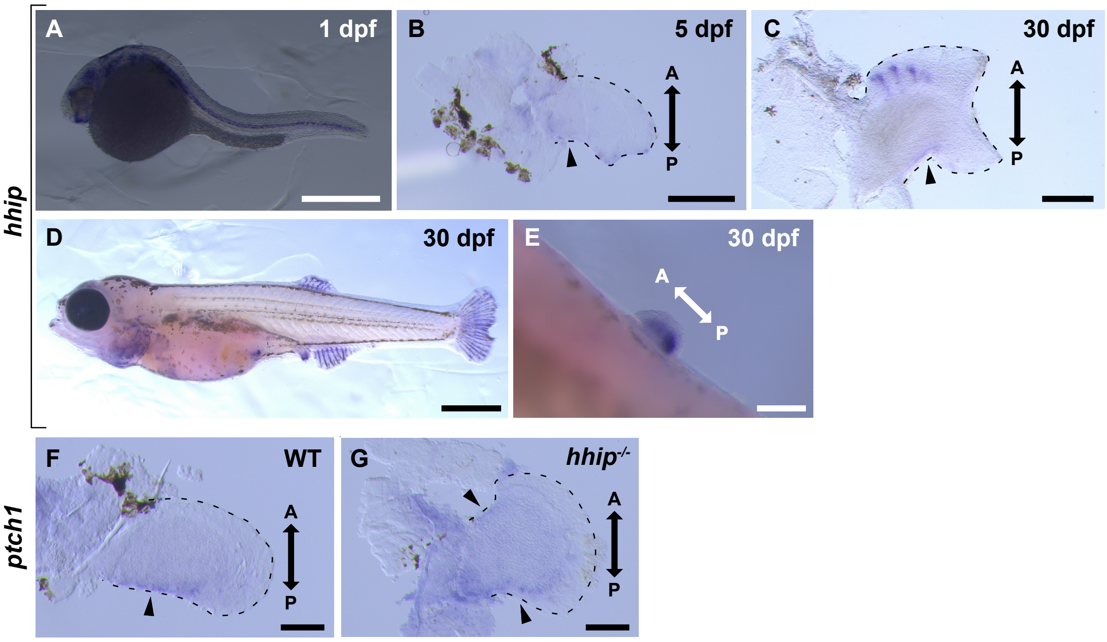


**Fig. S4. *hhip* expression pattern and the effect of *hhip* mutation on Hedgehog signaling.**

(A-E) *hhip* expression in zebrafish. (A) *hhip* expression in 1 dpf. *hhip* was expressed in the horizontal boundary cells of somites as reported previously (Abe et al., 2019; Koudijs et al., 2005). (B, C) *hhip* expression in pectoral fins. *hhip* was expressed in the posterior margin and fin rays. Black arrowheads indicate the *hhip* expression. (D) *hhip* expression in 30 dpf. In median fins, *hhip* was expressed in fin rays. (E) *hhip* expression in pelvic fins. (F, G) *ptch1* expression in *hhip*$^{-/-}$ and WT zebrafish. Black arrowheads indicate the *ptch1* expression. Scale bars: 100 µm (F, G); 200 µm (B, C, E); 500 µm (A); 1 mm (D).

Development • Supplementary information

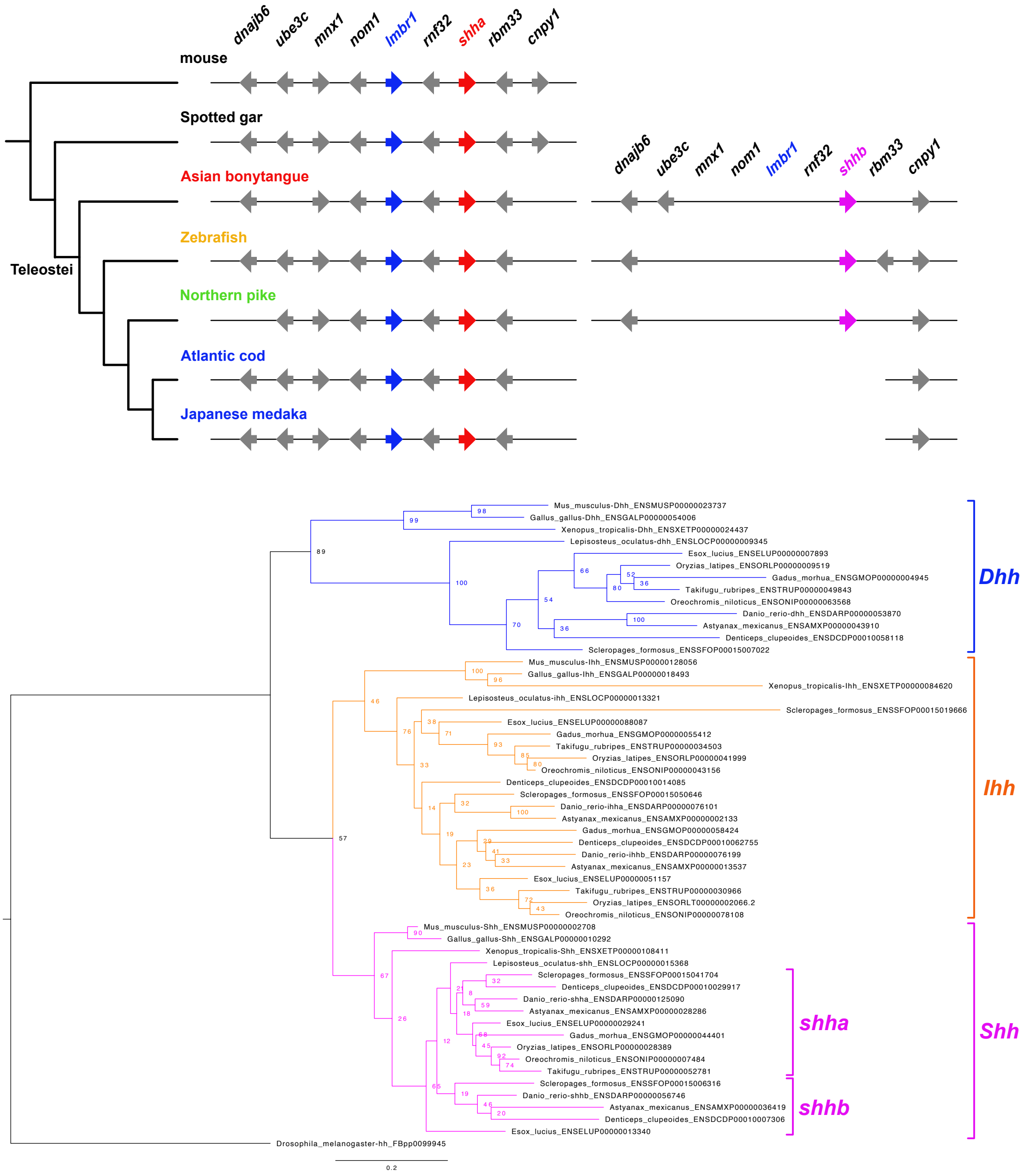


**Fig. S5. Comparison of Shh microsyntenic regions.**

Arrows indicate genes, with their directions representing the 5′ to 3′ orientation of the genes. Red arrows represent *shha* (or Shh genes in vertebrates except teleosts), while the magenta arrows represent *shhb*. Blue arrows represent *lmbr1*, which includes the ZPA regulatory sequence (ZRS) in the intron 5. Teleosts in Acanthomorpha have chromosomal recombination upstream of *cnpy1* and have lost *shhb* and the other microsyntenic genes. The values at the nodes of the phylogenetic tree represent bootstrap values.

Development • Supplementary information

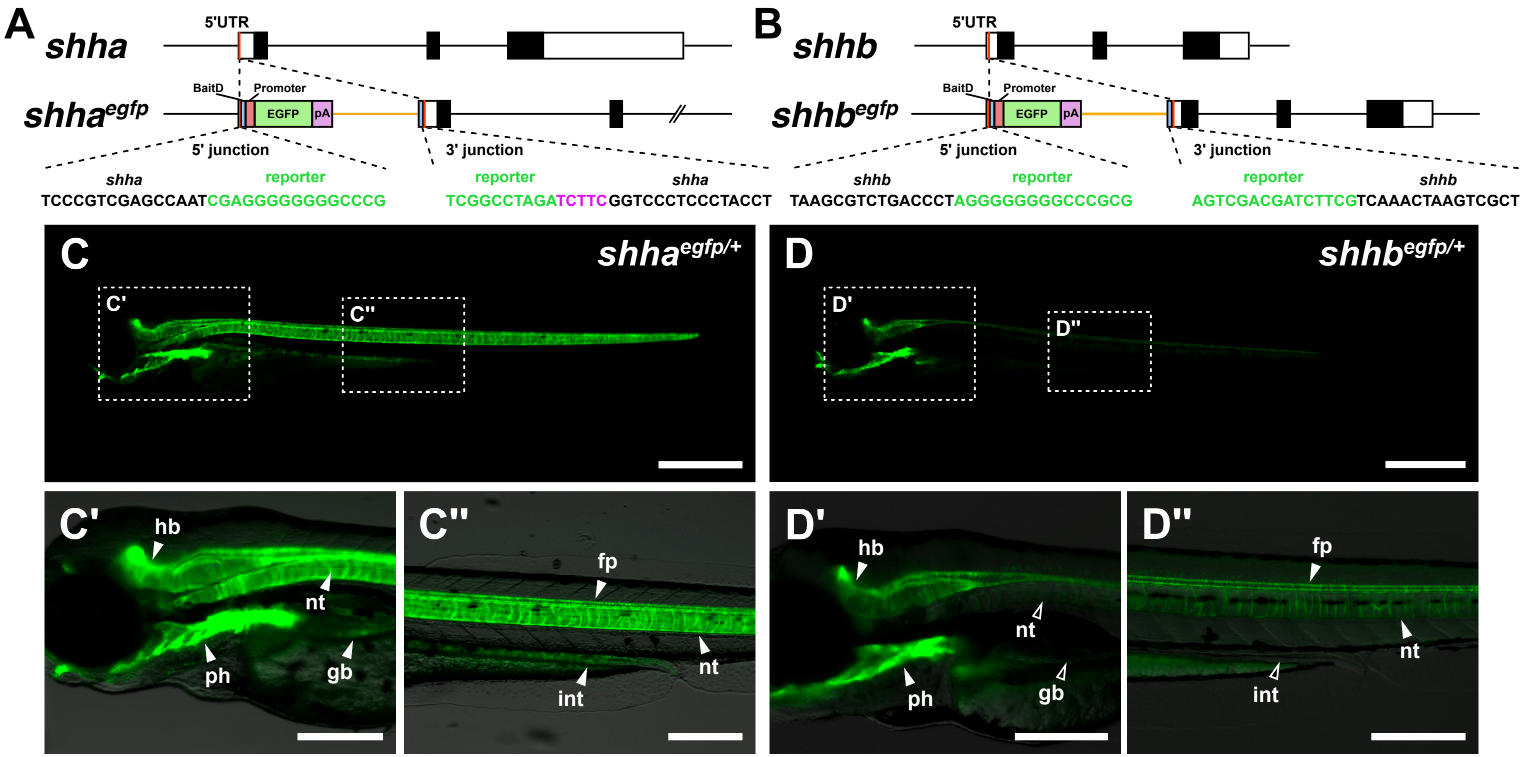


**Fig. S6. Zebrafish harboring *shha*$^{egfp}$ and *shhb*$^{egfp}$ knock-ins.**

(A, B) Sequences at the 5´ and 3´ junctions of *shha*$^{egfp}$ and *shhb*$^{egfp}$ zebrafish. Magenta letters represent a junk insertion via CRISPR-Cas9 editing. (C, D) Overall views of EGFP expression in 3 dpf of *shha*$^{egfp}$ (C) and *shhb*$^{egfp}$ zebrafish (D). Filled white arrowheads indicate EGFP expression and unfilled white arrowheads indicate no EGFP expression. fp, floor plate of neural tube; gb, gas bladder; hd, hindbrain; int, intestine; nt, notochord; ph, pharynx. These observations were made on 10 or more *shha*$^{egfp}$ and *shhb*$^{egfp}$ zebrafish larvae. Scale bars: 500 µm (C, D); 250 µm (C', C'', D', D'').

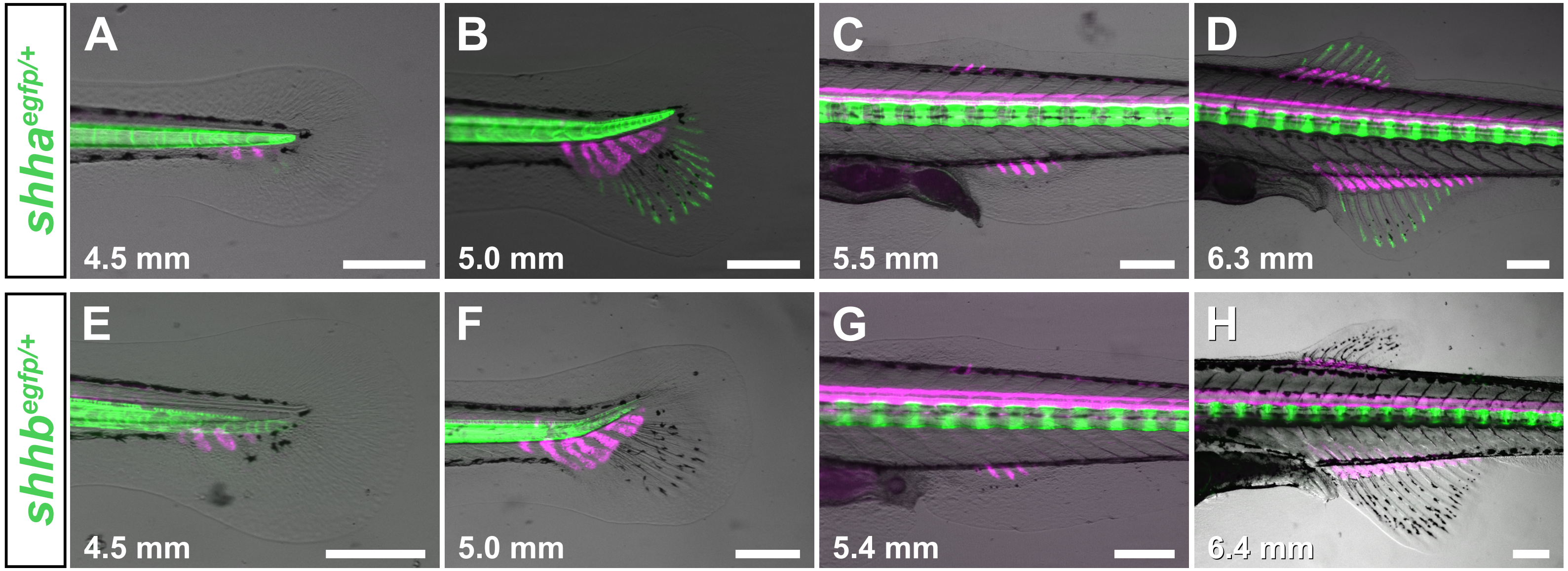


**Fig. S7. Expression of EGFP in *shha*$^{egfp}$ and *shhb*$^{egfp}$ zebrafish during median fin development.**

(A-D) EGFP expression during median fin development of *shha*$^{egfp}$ zebrafish. (A, B) Dorsal and anal fins. (C, D) Caudal fin. (E-H) EGFP expression during median fin development of *shhb*$^{egfp}$ zebrafish. (E, F: Dorsal and anal fins. G, H: Caudal fin) Magenta fluorescence indicates paired fin skeleton marked by *sox10:DsRed*. These observations were performed on 10 or more *shha*$^{egfp}$ and *shhb*$^{egfp}$ zebrafish larvae. The standard length of individuals (in mm) is shown in the bottom left of each panel. Scale bars: 250 µm (A-H).

Development • Supplementary information

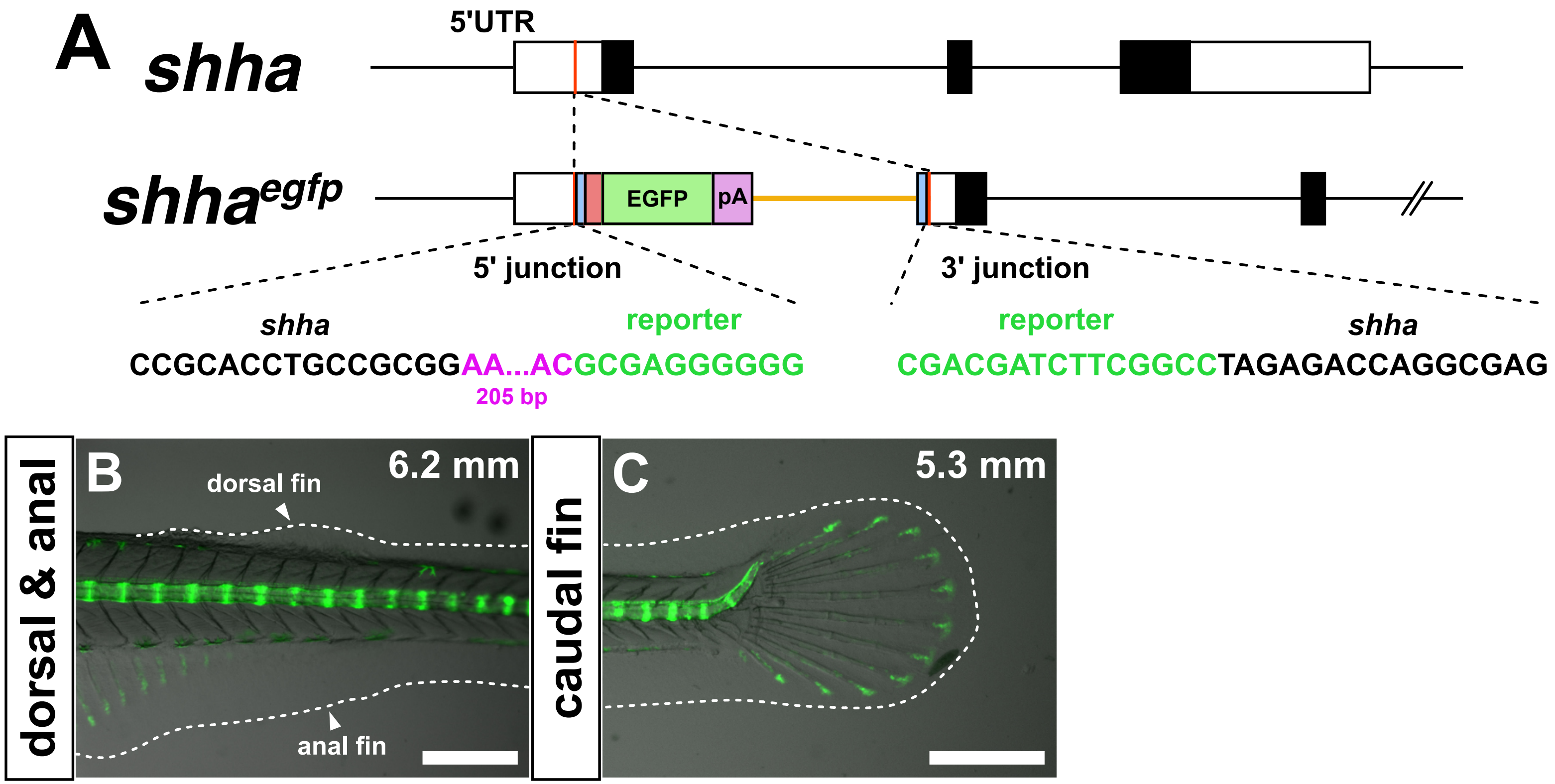


**Fig. S8. Japanese medaka harboring *shha*$^{egfp}$ knock-in.**

(A) Sequences at the 5′ and 3′ junctions of the *shha*$^{egfp}$ medaka. Magenta letters represent junk insertion via CRISPR-Cas9 editing. (B, C) EGFP expression in the dorsal and anal (B), and caudal fins (C) of *shha*$^{egfp}$ medaka. Dashed lines indicate the outline of fin folds. The standard length of individuals (in mm) is shown in the top right of panels (B, C). Scale bars: 500 µm (B, C).

Development • Supplementary information

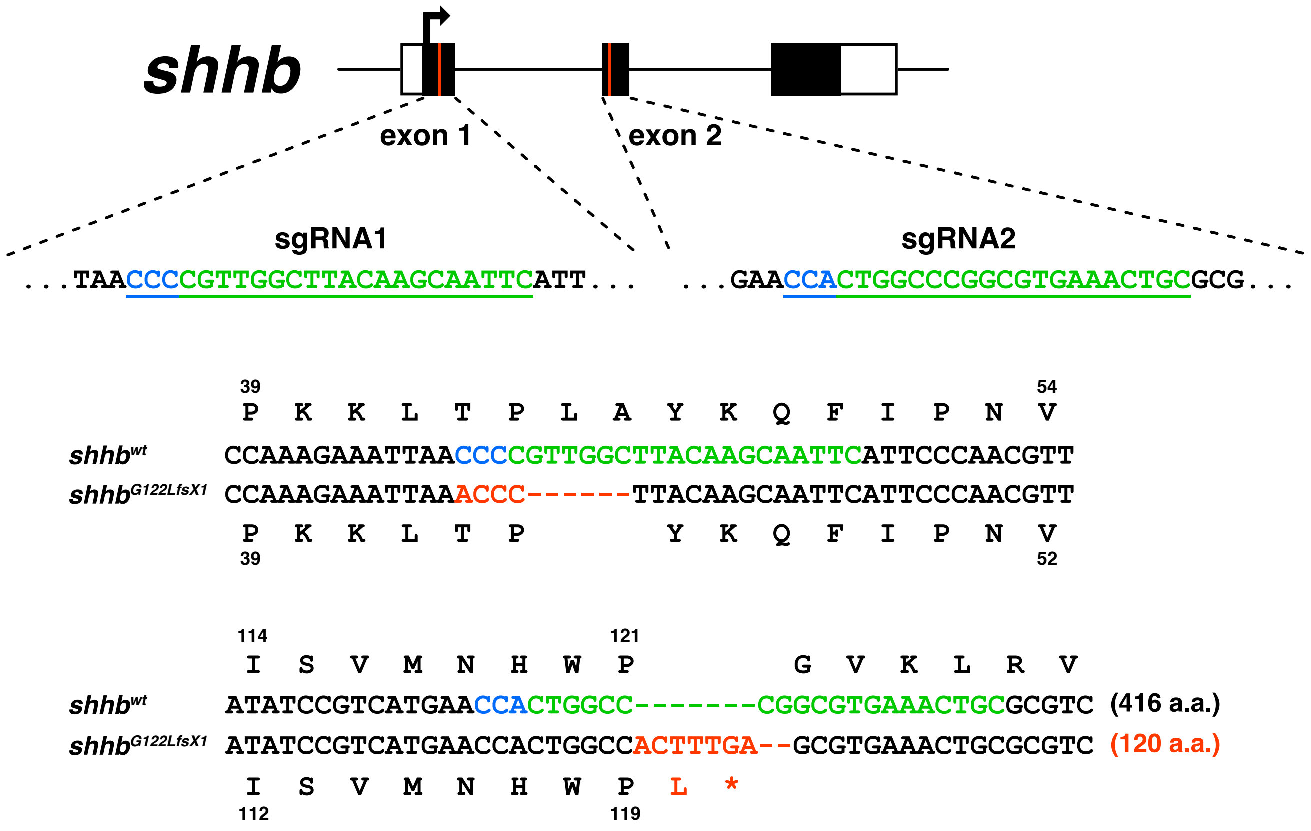


**Fig. S9. *shhb* mutation in zebrafish.**

The sequences in blue represent the protospacer adjacent motif (PAM), in green represent the recognition sequence for the sgRNA target, and in red represent the mutation site.

Development • Supplementary information

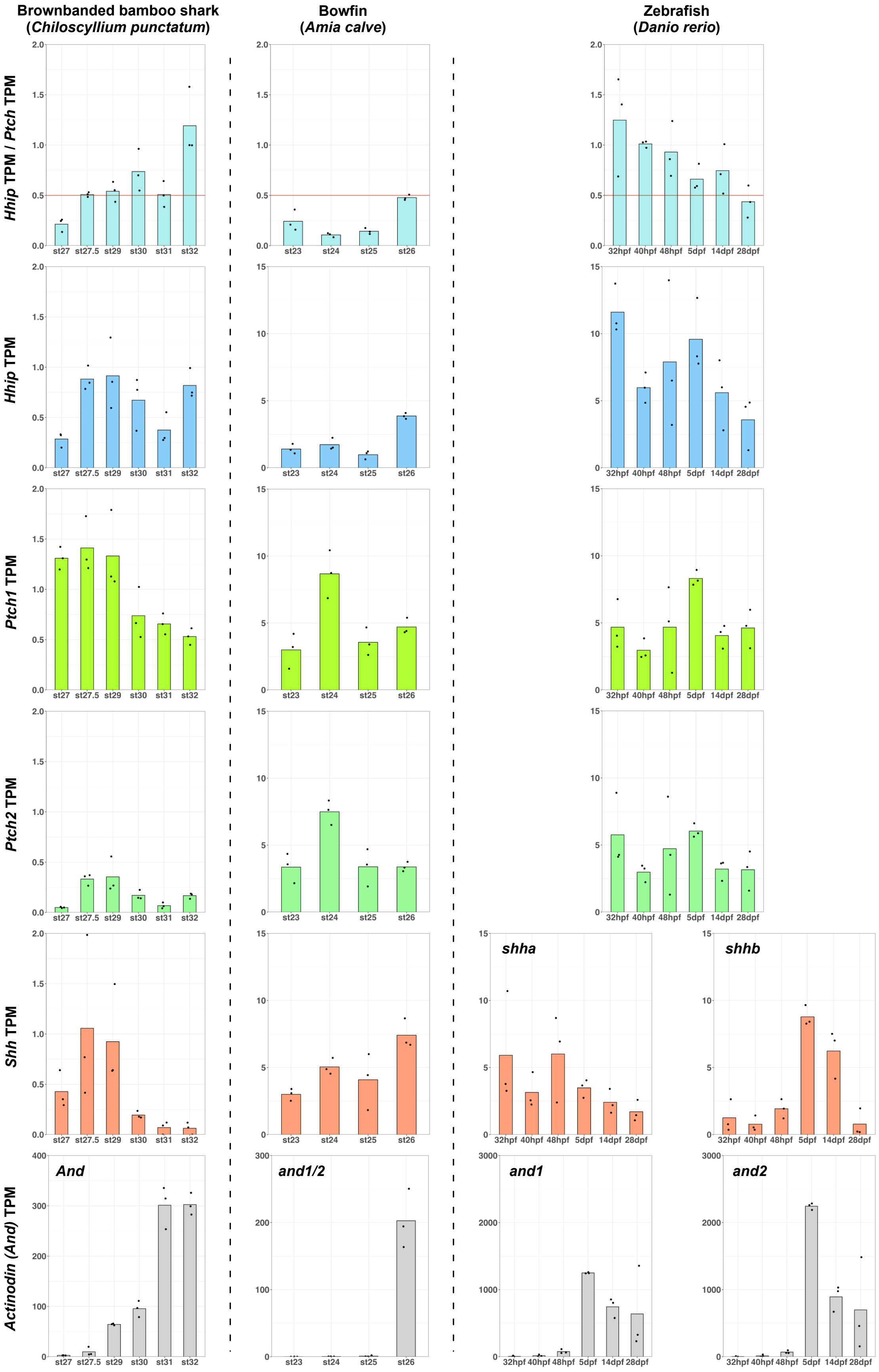
Brownbanded bamboo shark
(Chiloscyllium punctatum)
Bowfin
(Amia calve)
Zebrafish
(Danio rerio)
Hhip TPM / Ptch TPM
Hhip TPM
Ptch1 TPM
Ptch2 TPM
Shh TPM
Actinodin (And) TPM
shha
shhb
And
and1/2
and1
and2
st27 st27.5 st29 st30 st31 st32
st23 st24 st25 st26
32hpf 40hpf 48hpf 5dpf 14dpf 28dpf

**Fig. S10. RNA-seq analysis of *hhip* expression pattern in pectoral fins.**

The bar graphs, except those in the top row, show the expression levels (transcripts per million, TPM) at each developmental stage. The bar graph in the top row shows the *hhip* TPM divided by the sum of the *ptch1* and *ptch2* TPM for inter-species comparison. The *and* TPM distinguishes initiation of fin fold formation [brownbanded bamboo shark, from (st) 29; bowfin, from (st) 26; zebrafish, from 5dpf]. In the brownbanded bamboo shark and bowfin, the TPM value of *hhip* is initially below 0.5, converging around 0.5 after fin fold formation. However, in zebrafish, the *hhip* value starts well above 0.5 and converges around 0.5 after fin fold formation. The red lines represent the value of 0.5. Each dot on the graphs represents the value of each replicate. The RNA-seq data of pectoral fins were obtained from previously published brownbanded bamboo shark (Onimaru et al., 2021), bowfin (Thompson et al., 2021), and zebrafish (Kudoh et al., 2024) studies.

Development • Supplementary information

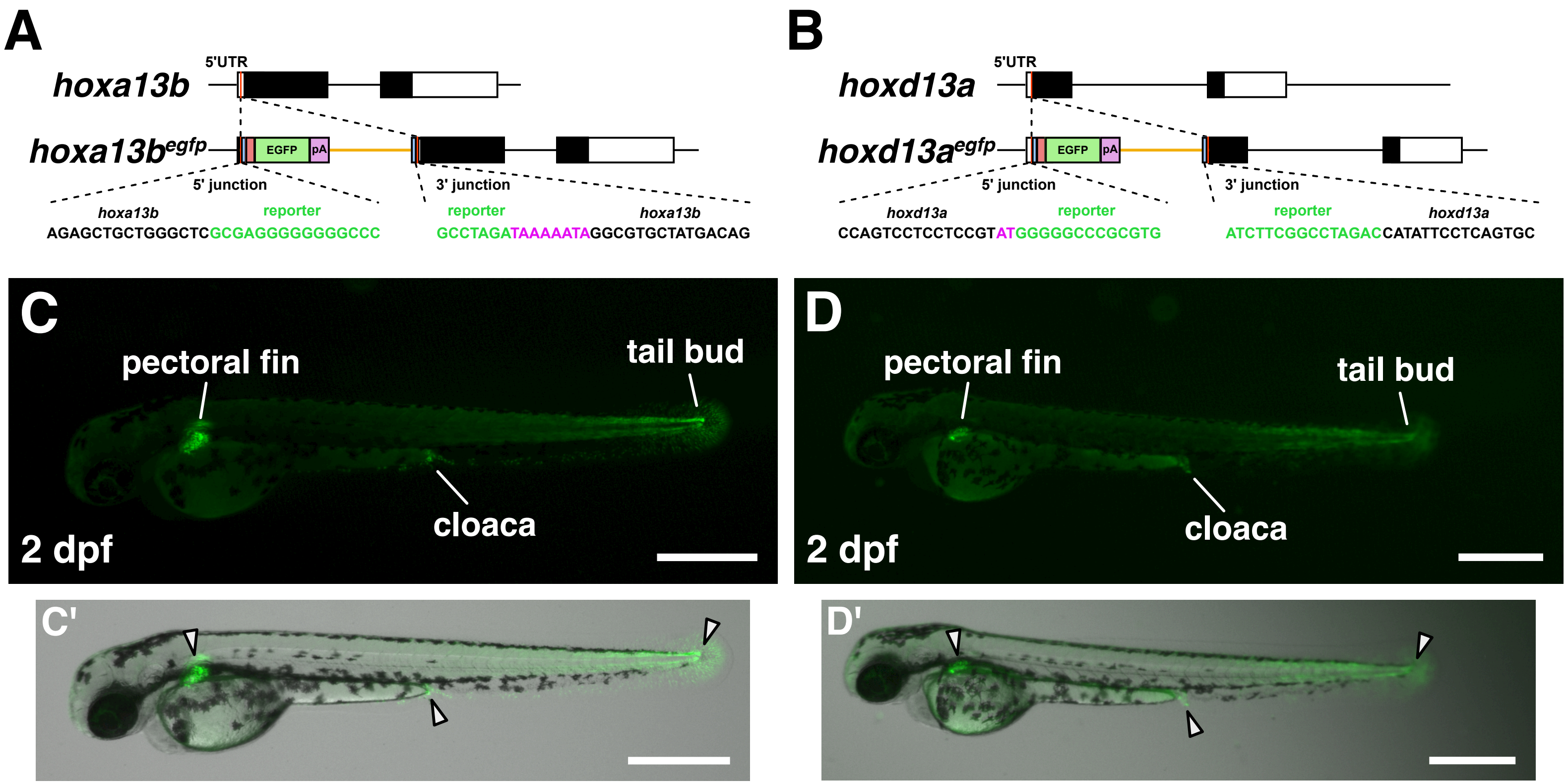


**Fig. S11. Zebrafish harboring *hoxa13b<sup></sup>*$^{egfp}$ and *hoxd13a*$^{egfp}$ knock-ins.**

(A, B) Sequences at the 5′ and 3′ junctions of the *hoxa13b*$^{egfp}$ and *hoxd13a*$^{egfp}$ zebrafish. Magenta letters represent a junk insertion via CRISPR-Cas9 editing. (C, D) Overall views of EGFP expression in 2 dpf of *hoxa13b*$^{egfp}$ (C, C') and *hoxd13a*$^{egfp}$ zebrafish (D, D'). White arrowheads indicate EGFP expression in the bright-field. These observations were made on 10 or more *hoxa13b*$^{egfp}$ and *hoxd13a*$^{egfp}$ zebrafish larvae. Scale bars: 500 µm (C, C', D, D').

Development • Supplementary information

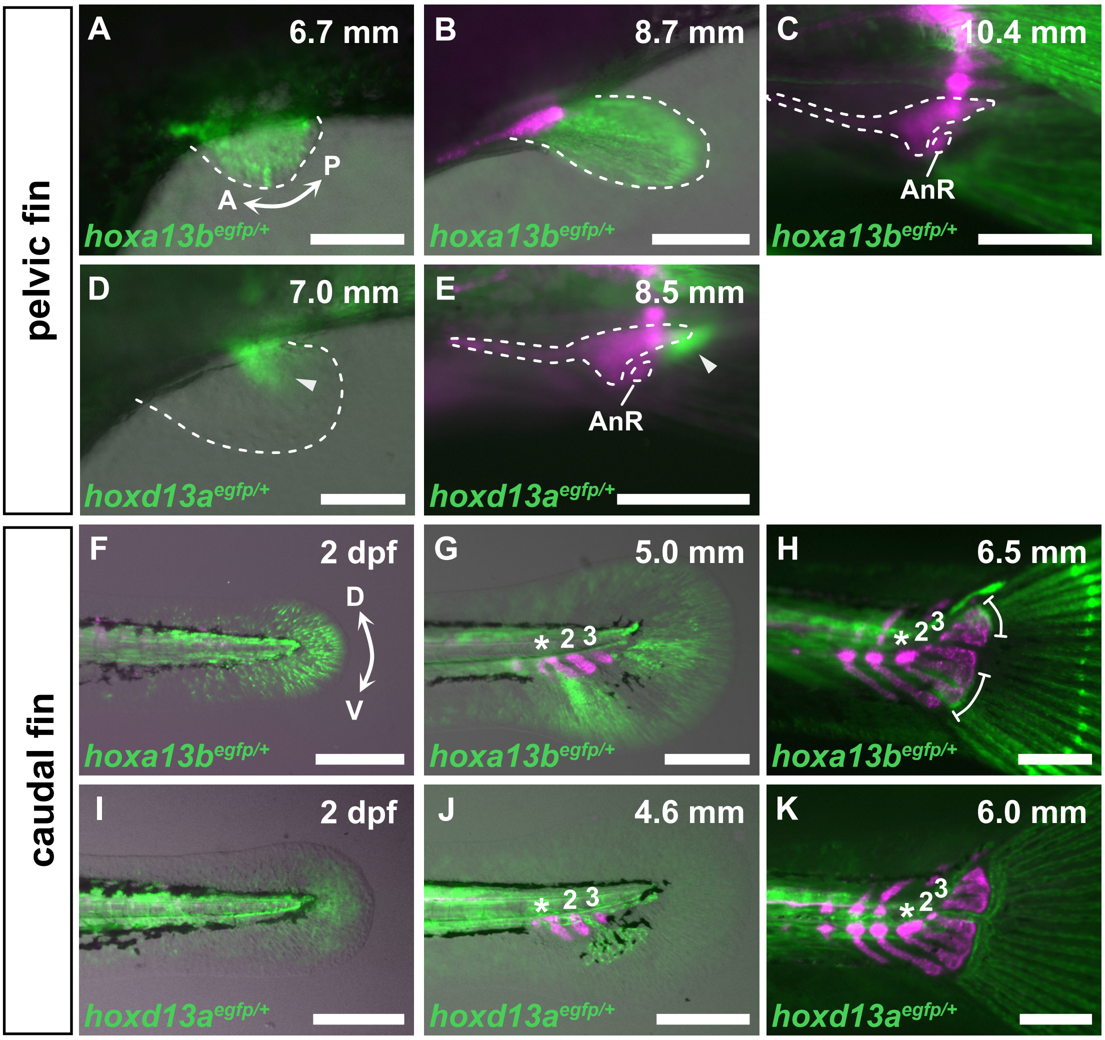


**Fig. S12. Expression of EGFP in *hoxa13b$^{egfp}$* and *hoxd13a$^{egfp}$* zebrafish during pelvic and caudal fin development of zebrafish.**

(A-E) EGFP expression during pelvic fin development of *hoxa13b$^{egfp}$* (A-C) and *hoxd13a$^{egfp}$* zebrafish (D, E). White arrowheads indicate EGFP expression. AnR, anterior large radial. (F-K) EGFP expression during caudal fin development of *hoxa13b$^{egfp}$* (F-H) and *hoxd13a$^{egfp}$* zebrafish (I-K). Numbers indicate 2nd and 3rd hypurals. The white asterisk marks the 1st ural vertebra. White bars indicate the range of EGFP expression. All observations were performed on more than 10 larvae at each developmental stage. Magenta fluorescence highlight paired fin skeleton marked by *sox10:DsRed*. Double arrows indicate the anterior (A)-posterior (P) axis and dorsal (D)-ventral (V) axis. The standard length of individuals (in mm) is shown in the top right of each panel. Scale bars: 125 μm (A, D); 250 μm (B, C, E-K).

Development • Supplementary information

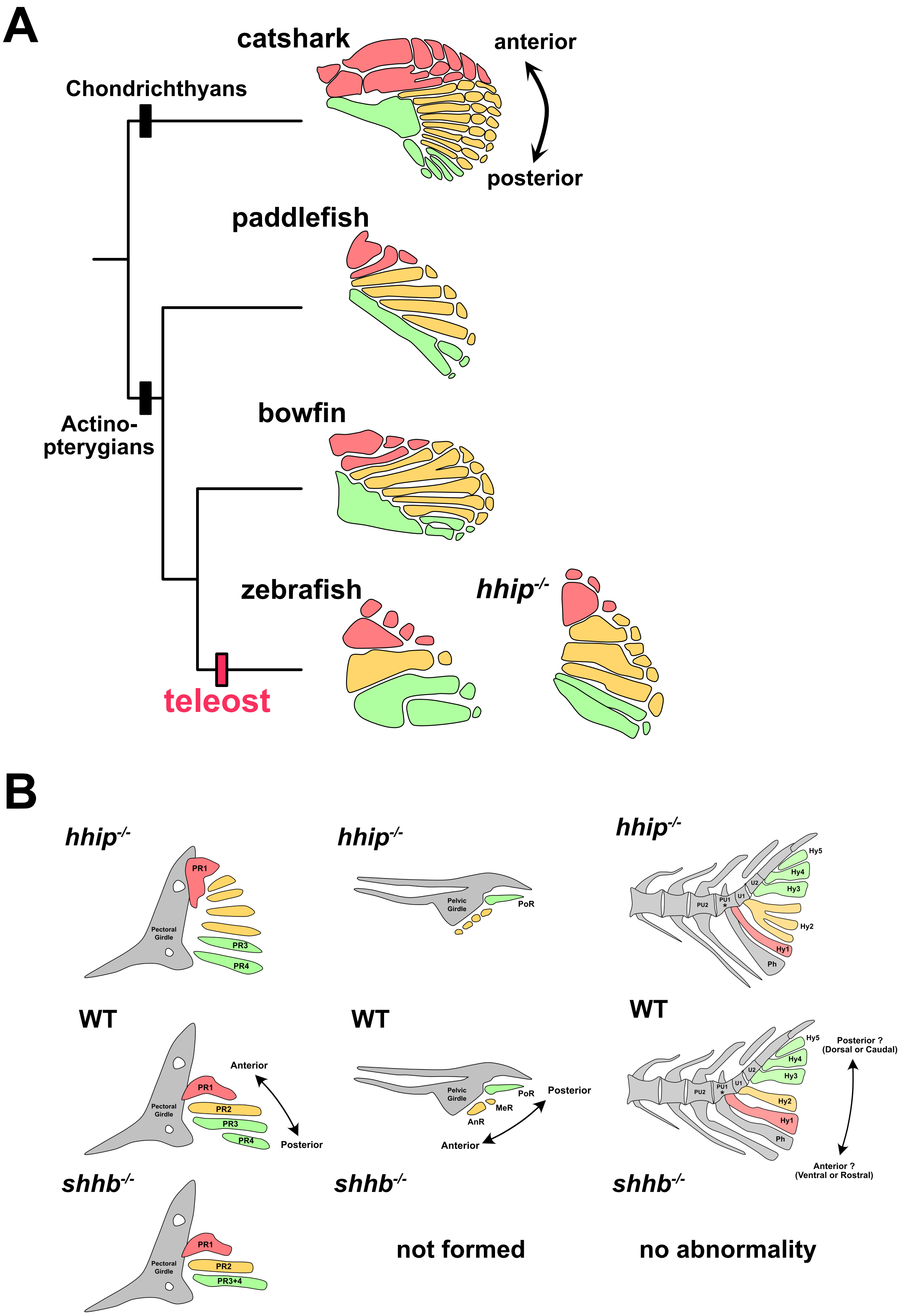


Development • Supplementary information

**Fig. S13. Hypothesis of fin regionality and homology.**

(A) Inter-species comparison of pectoral fins and (B) a comparison among pectoral, pelvic, and caudal fins in zebrafish. In pectoral fins, colors indicate the anterior region where neither *Hoxa13* nor *Hoxd13* is expressed (red), the median region where *Hoxa13* is expressed but *Hoxd13* is not expressed (orange), and the posterior region where both *Hoxa13* and *Hoxd13* are expressed (green) based on the expression pattern in zebrafish and those reported previously (Davis et al., 2007; Freitas et al., 2007; Tulenko et al., 2016). In pelvic and caudal fins, colors indicate hypothetical homology compared with pectoral fins. The skeletons were redrawn with modification from the original sources as follows: catshark (Onimaru et al., 2015; Onimaru et al., 2016), paddlefish (Davis et al., 2004; Mabee and Noordsy, 2004) and bowfin (Thompson et al., 2021).

**Table S1. CRISPR target sites**

| sgRNA name | CRISPR target site |
|---|---|
| zebrafish-*shha* knock-in | GAGGTAGGGAGGGACCTGATTGG |
| medaka-*shha* knock-in | ACAAAGCATTAAAGGCGGTCGG |
| zebrafish-*shhb* knock-in | GAGCGACTTAGTTTGACGGGAGG |
| zebrafish-*hoxa13b* knock-in | GAGCTGCTGGGCTCCATGTAGGG |
| zebrafish-*hoxd13a* knock-in | GCACTGAGGAATATGGACGGAGG |
| *hhip* mutagenesis site1 | TGCGCTCAGGAGATCCATAGTGG |
| *hhip* mutagenesis site2 | TGGAGAGGGAAGGCTTTGTGTGG |
| *shhb* mutagenesis site1 | GAATTGCTTGTAAGCCAACGGGG |
| *shhb* mutagenesis site2 | GCAGTTTCACGCCGGGCCAGTGG |
| *gli3* deletion site1 | GGGCTTGCTGCCAGTGCAGGAGG |
| *gli3* deletion site2 | GAGCGTATGTAGTCCATGTAGGG |

Development • Supplementary information

## Table S2. Primer sequences

### a) Mutation check primers

| Primer name | Forward sequence | Reverse sequence |
|---|---|---|
| *hhip*-HMA-site1 | CAAACTTGTCTGCCTAGCAGAAAAG | ACGCCAACAGGTTGTTGAAG |
| *hhip*-HMA-site2 | ATTGTGGAGATGGATCGCAG | CTTTGTACCAGCTTATGAATGTCCAG |
| *shhb*-HMA-site1 | TCCTTAAAGATGATATCGGG | TTATGGAAAACGAAGACACC |
| *shhb*-HMA-site2 | GGACAAGTTAAATTCGTTGG | TAAATGGTGACCATCCTCAT |
| *gli3*-deletion | AGGACTAAGGCCTCTGGCTG | ATCTCACTCTGCTGAATGAGTGG |

### b) Knock-in check primers

| Primer name | Forward sequence | Reverse sequence |
|---|---|---|
| zebrafish-*shha* | CGTCGCAGCCTCACACAGAC | TGCGACTGATAACGCATCGG |
| medaka-*shha* | ACAAAGCATTAAAGGCGGTCGG | GGCCGACCAAAACAAATTTGG |
| zebrafish-*shhb* | CACAGCAAAGCGAGAACAGG | GACCACAGGCTAATCCACAAGG |
| zebrafish-*hoxa13b* | CAGTTGGATCCTGACGCACG | CATCACCGGGTCAATCCAGC |
| zebrafish-*hoxd13a* | ACACCGGCTTGAGTGAGTCC | ACAGCTGCCCAATCACATGC |
| knock-in check | CGGTACCGCCAAAGTTGAGC | AATGCAGCTGGCACGACAGG |

Development • Supplementary information

**Table S3. Mutation list**

| **Mutant name** | **Mutation list** |
|---|---|
| *hhip*$^{H215RfsX1}$ | p.H215RfsX1, p.F242SfsX8 |
| *hhip*$^{F242SfsX8}$ | p.F242SfsX8 |
| *gli3*$^{\Delta}$ | p.A162YfsX33 |
| *shhb*$^{G122LfsX1}$ | p.L45_A46del, p.G122LfsX1 |

Red characters represent the first premature stop codon.

**Table S4. Probes used in in-situ hybridization**

| **Probe name** | **Forward sequence** | **Reverse sequence** | **Reference** |
|---|---|---|---|
| zebrafish-*hhip* | AATTTGTGCTCTTGTTAGCC | AGTGAGGTCCAGCAGGTAAG | Koudijs *et al*. 2005 |
| zebrafish-*ptch1* | AGGAACTCAAGTACACACGG | CTGGAACATGGTCTGAAAGG | This work |
