# Supplementary material for: Anterior-posterior constraint on Hedgehog signaling by hhip in teleost fin elaboration
Source: Development. 2024 Nov 18;151(22):dev202526. doi: 10.1242/dev.202526 (PMC11607692; doi:10.1242/dev.202526)
Supplement: Supplementary file 1 [file develop-151-202526_review_history.pdf]

Development

Anterior-posterior constraint on Hedgehog signaling by *hhip* in teleost fin elaboration

Yoshitaka Tanaka, Shun Okayama, Kohei Urakawa, Hidehiro Kudoh, Satoshi Ansai, Gembu Abe and Koji Tamura
DOI: 10.1242/dev.202526

Editor: James Briscoe

Review timeline

Original submission:	9 November 2023
Editorial decision:	18 December 2023
First revision received:	28 August 2024
Editorial decision:	25 September 2024
Second revision received:	9 October 2024
Accepted:	10 October 2024

Original submission

First decision letter

MS ID#: DEVELOP/2023/202526

MS TITLE: Fin elaboration via anterior-posterior regulation by Hedgehog signaling in teleosts

AUTHORS: Yoshitaka Tanaka, Shun Okayama, Satoshi Ansai, Gembu Abe, and Koji Tamura

I have now received all the referees' reports on the above manuscript, and have reached a decision. The referees' comments are appended below, or you can access them online: please go to BenchPress and click on the 'Manuscripts with Decisions' queue in the Author Area.

As you will see, the referees express considerable interest in your work, but have some significant criticisms and recommend a substantial revision of your manuscript before we can consider publication. The conclusion that modulation of hedgehog signalling during teleost evolution has reduced fin development will be of interest to our readers. An important issue to address in your revision is the data concerning the expression and interpretation of the Hox13 paralogs. Crossing the reporters to into hhip and shhb null lines would add to the study, but I recognise that this would take 6 months if the crosses have not already started. I also agree with the referees that a thorough editing of the text to improve the clarity of the writing would benefit your study.If you are able to revise the manuscript along the lines suggested, which may involve further experiments, I will be happy to receive a revised version of the manuscript. Your revised paper will be re-reviewed by one or more of the original referees, and acceptance of your manuscript will depend on your addressing satisfactorily the reviewers' major concerns. Please also note that Development will normally permit only one round of major revision. If it would be helpful, you are welcome to contact us to discuss your revision in greater detail. Please send us a point-by-point response indicating your plans for addressing the refereesâ€™ comments, and we will look over this and provide further guidance.

Please attend to all of the reviewers' comments and ensure that you clearly highlight all changes made in the revised manuscript. Please avoid using 'Tracked changes' in Word files as these are lost in PDF conversion. I should be grateful if you would also provide a point-by-point response detailing

© 2024. Published by The Company of Biologists under the terms of the Creative Commons Attribution License (https://creativecommons.org/licenses/by/4.0/).

how you have dealt with the points raised by the reviewers in the 'Response to Reviewers' box. If you do not agree with any of their criticisms or suggestions please explain clearly why this is so.

Reviewer 1

Advance summary and potential significance to field

In this manuscript Tanaka et al. investigate the role of hedgehog repressors on teleost fin development using zebrafish as their primary model system. They present mutants of secreted hedgehog antagonist hhip and the downstream associated transcription factor gli3 which is converted from repressor to activator when hedgehog signaling is active. The hhip data are most interesting and form the core of the manuscript while the gli3 data is presented more superficially as these do not give a strong phenotype probably due to redundancy with other gli gene family members (this in contrast to gli3 loss of function in Medaka which gives a very strong fin phenotype). Hhip mutants have been reported with fin phenotypes before in the Tubingen screen but as the authors remark the phenotype has never been investigated extensively therefore I do not think that this compromises novelty.

The hhip loss of function data are very interesting as these give a strong expansion of the pectoral fins. This phenotype is not just interesting from a developmental viewpoint, but also particularly from an evolutionary perspective.
Compared to the ancestral condition teleost fishes (which comprise over 99% of vertebrate aquatic diversity) have strongly reduced the endochondral part of their fins to four radial elements. The authors point out that the phenotype in the hhip mutants resembles the original ray finned fish condition and suggest that modulation of hedgehog signalling during evolution has reduced the middle part of the fins. This message is very well communicated in the final Fig.6.

In addition, the authors present GFP knockin lines of hoxa13b and hoxd13a to show anterior posterior patterning of the fins and they analyse the expression of shha and shhb using GFP knockin lines.

I am in general positive about the findings and I find the manuscript interesting and it could be considered for publication in Development. However there are also a number of issues and I think that these should be addressed.

Comments for the author

1. The authors appear to ignore what the expression of the hhip gene itself is. Is this ubiquitous? Or also developmentally regulated. It is unfortunate they did not produce a hhip GFP knockin line. I will not request this here, but some in situ data would be very helpful.
2. I find the paragraph associated with Fig. 3 quite problematic (line 150-168). Fig. 3 beautifully shows the expression of the hoxa13b and hoxd13a genes during fin development. I think these are the best and most conclusive data concerning the expression of these genes in fish fins thus far and I applaud the authors for providing these. They are very helpful in confirming standing issues relevant to the debate about the fin to limb transition, namely the possible anterior expression of hoxd13a (which doesn`t seem to be there) or exclusive expression of hox13 these in the dermal fin compartment (it also appears in the distal radials). The problem is that the way the lines are presented in the manuscript they very poorly serve the story the authors bring and I frankly see no good reason for their inclusion. The only conclusion that the authors adhere to these data is that pectoral and pelvic fin radials are patterned along their anterior proximal axis by hedgehog responsive genes. This is of course very well-known and the authors cite much of the relevant literature in the discussion. What would be interesting and new is to cross these GFP lines into hhip and shhb null fishes and see if and how their expression changes. This could for instance further confirm whether the supernumerary radial divisions in the hhip mutants really occur in the original radial 2/3 part of the fins. Since this appears a logical venue of experimentation, I can imagine that the authors already have initiated such crosses, and I would very much encourage them to include this into the paper. As said, Fg.3 as is, is not logically incorporated into the manuscript.

© 2024. Published by The Company of Biologists under the terms of the Creative Commons Attribution License (https://creativecommons.org/licenses/by/4.0/).

3. In the paragraph from line 170-211 the authors make the point that in contrast to shha, the shhb paralog is expressed during late fin development when the endochondral disk separates into radials and that the single Medaka shh paralog recapitulates the combines expression of zebrafish shha and shhb. The authors use this argument to put extra weight on the function of shhb for the phenotype they observe. I am really not sure this is a logical construction. In the supplement the authors report an enlarged early endochondral disk for the hhip mutants. The expanded fin phenotype therefore appears to emerge early and could hence be the consequence of both shha and shhb signaling. That the radial divisions only emerge during late development might have nothing to do with the genes that are still active at the moment of these divisions. More likely the supernumerary radials are a manifestation of a bigger disk, which can accommodate a larger number of divisions (activity of a Turing mechanism?). This is also consistent with the reduced number of radials in mutants of the ZRS and shadow enhancers associated with the shha locus (Letelier et al. 2018). The observations on shhb are still very interesting because they demonstrate that modulation of hedgehog strength by changing the shh copy number decreases fin size. I would strongly suggest the authors to emphasize this part of the message and focus less on whether the shh genes are expressed early or late because I am really not sure whether this matters (and this is also not shown).
4. The authors put a strong emphasis on the comparative/evolutionary context of their findings. Their phrasing in the manuscript is very careful and conjecturing because of course they do not actually show that hedgehog signaling is more active in basal fishes with elaborated fins. Although I thus think that their claims are appropriate, the evolutionary message will probably be the part of the paper that sticks with most readers (also because of the very nice homology drawings in Fig. 6). Therefore, if the authors have any data on basal species such as gar, sturgeon or shark indicating for instance lower levels of hhip expression they would be strongly encouraged to include these in the manuscript.
5. Basal fishes also tend to have more caudal fin radials. Given the caudal fin phenotype in the hhip mutant the authors might want to include a line or two on this in the comparative paragraphs.
6. The authors might want to better highlight the effect on the anal fin in comparison to other hedgehog mutants. Its disappearance instead of being enhanced with increased hedgehog signaling is very interesting as it confirms prior work with gli3 and zrs mutant fishes which show a similar bizarre difference in hedgehog sensitivity between the anal and other fins. (But note that cichlids in contrast to other fish appear to have a ZPA in the anal fin (Höch et al. 2021) making the comparative context of these observations rather mysterious and difficult to understand.)

<u>Reviewer 2</u>

Advance summary and potential significance to field

See the comments below.

Comments for the author

This manuscript investigates the function of the Hedgehog signaling in zebrafish paired fins and its involvement in teleost fin evolution. The reduction of radials and loss of the metapterygium are dominant themes in teleost evolution, yet their genetic underpinnings remain largely unknown. Combining analyses of several genetic knockout and transgenic zebrafish, Tanaka et al. showed that:

1. The Hedgehog signaling regulates the number of proximal radials

2. The spatially distinct expression domains of hoxa13 genes

3. The expression patterns of shh genes during radial bone development in zebrafish and medaka

© 2024. Published by The Company of Biologists under the terms of the Creative Commons Attribution License (https://creativecommons.org/licenses/by/4.0/).

All of the presented data are novel and of exceptional quality, illuminating the genetic mechanisms underlying the reduction of proximal radials in teleost lineages. I highly recognize the time-consuming effort of Tanaka et al. to develop all of the presented knockout and transgenic lines, which would be a great resource for the community. Finally, some paragraphs in the Discussion section are superb; for instance, the possible linkage of the evolutionary processes of paired and unpaired fins in teleosts is an exhilarating concept that should be pursued in future studies. As such, the hypotheses of fin skeletal evolution presented throughout the manuscript are scientifically pleasant and contemplating. Overall, Tanaka et al. generated an exceptional series of data and hypotheses that dissect the developmental and evolutionary functions of the Hedgehog signaling in teleost fins.

While I fully appreciate the quality and novelty of this manuscript, this manuscript needs major improvements in the integration of all experimental results and scientifically accurate writing. Despite the excellence of each experimental result, they seem to not be functionally integrated well into the major goal of this paper: How are hhip, shh, and hox13 genes related to each other in the middle radial patterning? I also had a difficult time comprehending many writings due to scientific obscureness. The authors should thoroughly revise the text for scientific clarity. Finally, grammatical errors and typos are found throughout the manuscript. For further consideration of this manuscript, I highly recommend that the authors comprehensively improve the experimental scheme, the integration of all experiments, and text writing. See the detailed comments below.

Majors:

*In the paired fins of hhip knockout zebrafish, the second radial is divided and becomes multiple radials, recapitulating the morphological features of basal actinopterygians and chondrichthyans. As Hhip is a direct binding inhibitor of Hedgehog proteins, we would expect that hedgehog ligand knockout zebrafish also show defects around the second radial. However, Fig.5 exhibits that Shhb is functional in posterior pectoral fin morphogenesis. How do the authors explain this counter-intuitive difference in the affected areas between the pectoral fins of these knockout fish? Is the upregulation of Hedgehog signaling in hhip knockout fish caused by shha, not shhb? It would be great if the authors investigated active hedgehog signaling in developing fins, such as by ISH of the hedgehog signaling reporter gene (e.g., ptch). Also, is it possible to treat hhip mutant embryos with a chemical inhibitor of Hedgehog signaling and compare ptch expression patterns between wildtype, hhip mutant, hhip mutant treated by the inhibitor fish? It is highly recommended to explore the molecular underpinnings of the phenotypic discrepancy between hhip and shhb knockout zebrafish. The currently presented data, figures, and story are not integrated to substantiate the mechanisms that the authors assume.

*Hoxa13b and d13a expression patterns were monitored by EGFP knock-in zebrafish. These observations identified the three distinct domains with diverse hox expression patterns. While this finding could be informative to understand hhip mutant fish phenotype (and more generally, pectoral fin development, for sure), I'm wondering how this finding is better integrated into the main story of the manuscript. Is hox13 expression, particularly hoxa13b, regulated by the Hedgehog signaling? Is hoxa13b expression upregulated in hhip mutant fish? Ahn and Ho utilized Cyclopamine to reduce Hedgehog signaling and observed the disappearance of hoxa13a expression (Development, 2008). It is highly recommended that the expression level of hoxa13 in hhip mutant fish be analyzed.

*In Fig. 6, the three colors were applied to indicate three different hox expression domains in pectoral fins.

Although this is a novel attempt to comprehend developmental fin regionality, we don’t know exactly which bones express which hox genes in catsharks, paddlefish, and bowfins. These color codes are misleading and should be presented as a hypothesis.

Minors:

Line 69-71: The current writing says, "Shh signaling reduces the number of radials in them," which is not what the authors meant, I suppose.

© 2024. Published by The Company of Biologists under the terms of the Creative Commons Attribution License (https://creativecommons.org/licenses/by/4.0/).

Line 113: "Regional-specific" should be "Region-specific".

Line 125-127: I don't understand the writing and logic. This sentence needs to be more elaborated or clarified to explain how counterparts were identified between wildtype and mutant fish.

Line 168: A hyphen is unnecessary.

Line 172-173: Can this sentence be rephrased like "The high expression levels of hoxa13a/d13a in the distal region of posterior radials……"? I don't see the data that support the current writing, such as "The increased expression" or "with increasing proximity to the posterior region" in Fig. 3.

Line 174: I guess the authors meant "region-specific expansion of endochondral radials" by "region-specific expansion".

Line 198: "will have lost". Did the author mean "might have lost"?

Line 222: "regulated" should be "regulates."

Line 279: "these results" – did the author mean "current results" or "our results"? It's unclear what the authors refer to.

Line 292 should be like, "The first discrepancy is the repression mechanism of Hedgehog signaling."

Line 301: Remove "in"

Line 303: "regulated" should be "regulate(s)."

Line 312: "shhb remains a paired fin enhancer…..". Shhb is not an enhancer.

Line 326-327: If my understanding is correct, this sentence must be elaborated more for scientific accuracy.

For example, does "augmented Hedgehog signaling" mean "augmented Hedgehog signaling from basal actinopterygians to teleosts" or what? What the authors meant is scientifically unclear.

Reviewer 3

Advance summary and potential significance to field

The paper reports the generation of several CRISPR generated gene knockin reporter lines and mutants in the shh signalling pathway and there use to determine the role of shh signalling and modulation of shh signalling in zf fin formation and the evolution of fin adaptations during the evolution of fishes.

Comments for the author

There is an impressive number of CRISPR generated mutants described in the results section but how they contribute to the overall message of the paper is not always clear

Results:

Line 95 "In the pectoral fins of hhip-/- mutants, the pectoral girdle position was shifted laterally compared to that of WT zebrafish, which was similar to the position in chondrichthyans and basal actinopterygians (Fig. 1A, B" This is not clear from the results shown

114 "Fishes that retain the ancestral state of paired fins show an increase in radials along the AP axis during development (Davis et al., 2004; Mabee and Noordsy, 2004; Onimaru et al., 2016). To

© 2024. Published by The Company of Biologists under the terms of the Creative Commons Attribution License (https://creativecommons.org/licenses/by/4.0/).

identify the regionality of these increases, we examined fin skeletons along the AP axis during the development of hhip-/- mutants (Fig. 2).”
The logic of this statement is unclear/muddled. The ancestral state is to have more radials and thus in teleost there has been a decrease of radials…

Discussion:
The discussion sections contains some significant speculation and some misstatements/lack of clarity that requires some editing to correct.

Over interpretation. The results do not “explain the developmental change in the morphological evolution form the ancestral form to the teleost-specific derived form”
At best, they can support a potential developmental mechanism.

IT is not clear that the zf hhip mutant fins “largely correspond” to the endochondral bone formed in some chondrichthyans and basal actinopterygians.

Minor

The title of the paper is vague and could perhaps be modifed to more explicitly state the core results of the manuscript.

No explanation is offered for the CRISPR induced gli3 mutant not replicating published zf gli3 mutants

223 Surprisingly, pelvic fin skeleton, including pelvic girdles, was absent in almost all shhb mutants (Fig. 5C).
This is not discussed further..why would an absence of fin be the predicted phenotype of absence of shh signalling in the fin?

Minor typographical

Line 280 “Although neither shha nor shhb expression were detected in the endochondral primordium of the caudal fin skeleton (Fig. S7A, B, E, F), Shh secreted by other organizers, such as the notochord, regulate hypural development (Hadzhiev et al.,2007).”
Incomplete second clause. Meaning is therefore unclear

309 “has become a kind of gene desert” term “kind of” should be removed
310 “located conserves the lmbr1 paralog” is unclear
133 “This developmental observation revealed an abnormality of the pelvic fin skeleton in hhip-/- mutants (Fig. 2G-L)”
A new paragraph starts with this statement so it is unclear what “This developmental observation” relates to

310 “ considering that the expression pattern of Shh genes in paired fins of zebrafish and medaka, we speculate that zebrafish shhb remains a paired fin enhancer homologous to the ZRS of shha in the gene desert and achieves the fine-tuning regulation of Shh expression, while in medaka, the loss of one of the Shh genes simplifies such regulation”
Requires edits and modification to correct errors and to improve clarity

314“This diversity in Hedgehog signaling regulation” This has not actually been shown but is speculated on

---

First revision

Author response to reviewers' comments

Point-by-point response

© 2024. Published by The Company of Biologists under the terms of the Creative Commons Attribution License (https://creativecommons.org/licenses/by/4.0/).

Dear Editor

Thank you for considering our manuscript. As per the Editorial and Reviewers' suggestions, the manuscript has been thoroughly revised. We believe the reviewers comments have significantly helped us in improving the manuscript. We highly appreciate the constructive suggestions.

As suggested, whole main text and almost all figures/legends have been revised.
Before we respond to reviewer comments, we reported two revised points on our own initiative below.

- A phylogenetic tree of Hedgehog ligands in Fig. S5 has been added as phylogenetic identities of *shha* and *shhb* were not supported in the previous version.

- We retracted the suggestion about the number of *gli2*. We stated that the *gli2* in Japanese medaka Hd-rR was existed in one copy, based on the Japanese medaka Hd-rR genome assembly uploaded on Ensembl (ASM223467v1). We have carefully reanalyzed it with a new genome assembly (Hd-rR_v2.2.4: http://viewer.shigen.info/medaka/blast.php) and detected *gli2a* of Japanese medaka. Therefore, we deleted the following sentences from the manuscript.
"This difference may be related to the number of Gli2 genes serving as GLI repressors (Bowers et al., 2012; Karlstrom et al., 2003). For most teleosts, including zebrafish, two Gli2 genes remain, but Japanese medaka (Oryzias latipes) has lost gli2a and only gli2b remains."

Please see below our detailed point-by-point responses.

(Italics are reviewer and editor remarks to the authors)
Editor decision
As you will see, the referees express considerable interest in your work, but have some significant criticisms and recommend a substantial revision of your manuscript before we can consider publication. The conclusion that modulation of hedgehog signalling during teleost evolution has reduced fin development will be of interest to our readers. An important issue to address in your revision is the data concerning the expression and interpretation of the Hox13 paralogs. Crossing the reporters to into hhip and shhb null lines would add to the study, but I recognise that this would take 6 months if the crosses have not already started. I also agree with the referees that a thorough editing of the text to improve the clarity of the writing would benefit your study. If you are able to revise the manuscript along the lines suggested, which may involve further experiments, I will be happy to receive a revised version of the manuscript.

Response: We agree that we need to revise the entire manuscript, especially the *Hox13* paralog part. Therefore, we have revised the entire manuscript and clarified the two main findings: (i) the mechanism of radial reduction and restriction by Hedgehog signaling in teleosts, and (ii) the genetic comparison between zebrafish and basal species. We moved the *Hox13* paralog section to the end of the Results because it pertains to point (ii), and its original placement disrupted the flow of point (i). Additionally, we performed a cross between *hhip*$^{-/-}$ and *hoxa13b*^{egfp} zebrafish to demonstrate that the increased radials in the pectoral fins of *hhip*$^{-/-}$ zebrafish are located in the medial region with *hoxa13b* expression.

Response to Reviewer 1
Reviewer comment 1-1
1. The authors appear to ignore what the expression of the hhip gene itself is. Is this ubiquitous? Or also developmentally regulated. It is unfortunate they did not produce a hhip GFP knockin line. I will not request this here, but some in situ data would be very helpful.

Response 1-1: Thank you for the critical comment. We conducted in situ hybridization (ISH) analysis with *hhip*. The ISH showed that *hhip* expression is developmentally regulated and specific to the posterior margin of pectoral fins. We have added this data (Fig. S4) and the text as follows.

Page 6 Lines 118-127: " Next, to identify the role of *hhip* in fin development, we examined the expression pattern of *hhip*. The expression pattern of *hhip* in the early stages has been previously reported: *hhip* is significantly expressed in the entire pectoral fin bud at 48 hours post fertilization

© 2024. Published by The Company of Biologists under the terms of the Creative Commons Attribution License (https://creativecommons.org/licenses/by/4.0/).

(hpf) (Koudijs et al., 2005). We observed that *hhip* was only slightly expressed in the posterior margin of the pectoral fin after 5 days post-fertilization (dpf) (Fig. S4B, C). In median fins, *hhip* was highly expressed in the fin rays, but not in the endochondral regions (Fig. S4D). In pelvic fin buds, *hhip* was significantly expressed (Fig. S4E), similar to what was observed in pectoral fin buds at 48 hpf. These results indicated that *hhip* works specifically during the early development of the paired fins in zebrafish."

Reviewer comment 1-2

2. I find the paragraph associated with Fig. 3 quite problematic (line 150-168). Fig. 3 beautifully shows the expression of the hoxa13b and hoxd13a genes during fin development. I think these are the best and most conclusive data concerning the expression of these genes in fish fins thus far and I applaud the authors for providing these. They are very helpful in confirming standing issues relevant to the debate about the fin to limb transition, namely the possible anterior expression of hoxd13a (which doesn`t seem to be there) or exclusive expression of hox13 these in the dermal fin compartment (it also appears in the distal radials). The problem is that the way the lines are presented in the manuscript they very poorly serve the story the authors bring and I frankly see no good reason for their inclusion. The only conclusion that the authors adhere to these data is that pectoral and pelvic fin radials are patterned along their anterior proximal axis by hedgehog responsive genes. This is of course very well- known and the authors cite much of the relevant literature in the discussion. What would be interesting and new is to cross these GFP lines into hhip and shhb null fishes and see if and how their expression changes. This could for instance further confirm whether the supernumerary radial divisions in the hhip mutants really occur in the original radial 2/3 part of the fins. Since this appears a logical venue of experimentation, I can imagine that the authors already have initiated such crosses, and I would very much encourage them to include this into the paper. As said, Fg.3 as is, is not logically incorporated into the manuscript.

Response 1-2: We agree with the comment. Therefore, we crossed *hhip*$^{-/-}$ and *hoxa13b*^{egfp} and observed *hhip*$^{-/-}$; *hoxa13b*$^{egfp/+}$ zebrafish. The increased radials of the pectoral fins in *hhip*$^{-/-}$ zebrafish are included in the medial region with *hoxa13b* expression. We conducted limited experiments due to the fragility of *hhip*$^{-/-}$ zebrafish. The *hhip*$^{-/-}$ was so weak that it was difficult to raise them to a sufficient. The weakness of *hhip*$^{-/-}$ only allowed us to harvest *hhip*$^{-/-}$; *hoxa13b*$^{egfp/+}$ double transgenic fish. We have included this data (Fig. 6D-F) and additional text as follows.
Page 10-11 Lines 293-301: "In *hhip*$^{-/-}$ zebrafish, *hoxa13b*^{egfp} was expressed in the distal margin, but EGFP was highly expressed in posterior cells compared to WT zebrafish (Fig. 6D, the arrowhead). Throughout the subdivision, the *hoxa13b*^{egfp} was expressed not only on the distal margin of the most posterior radials equivalent to the 3rd and 4th radials in WT zebrafish but also on the distal margin of the increased radials equivalent to the 2nd radial in WT zebrafish (Fig. 6E, F). These results suggest that the pectoral fin of zebrafish is regionalized by distinct patterns of *hoxa13* and *hoxd13* expression, and that the number of radials in *hhip* mutants increases in the median region where *hoxa13b* is expressed."

Reviewer comment

3. In the paragraph from line 170-211 the authors make the point that in contrast to shha, the shhb paralog is expressed during late fin development when the endochondral disk separates into radials and that the single Medaka shh paralog recapitulates the combines expression of zebrafish shha and shhb. The authors use this argument to put extra weight on the function of shhb for the phenotype they observe. I am really not sure this is a logical construction. In the supplement the authors report an enlarged early endochondral disk for the hhip mutants. The expanded fin phenotype therefore appears to emerge early and could hence be the consequence of both shha and shhb signaling. That the radial divisions only emerge during late development might have nothing to do with the genes that are still active at the moment of these divisions. More likely the supernumerary radials are a manifestation of a bigger disk, which can accommodate a larger number of divisions (activity of a Turing mechanism?). This is also consistent with the reduced number of radials in mutants of the ZRS and shadow enhancers associated with the shha locus (Letelier et al. 2018). The observations on shhb are still very interesting because they demonstrate that modulation of hedgehog strength by changing the shh copy number decreases fin size. I would strongly suggest the authors to emphasize this part of the message and focus less on whether the shh genes are expressed early or late because I am really not sure whether this matters (and this is also not shown).

© 2024. Published by The Company of Biologists under the terms of the Creative Commons Attribution License (https://creativecommons.org/licenses/by/4.0/).

Response 1-3: We agree with "*More likely the supernumerary radials are a manifestation of a bigger disk, which can accommodate a larger number of divisions (activity of a Turing mechanism?).*", and therefore, focused on the growth of the endochondral disc in the revised manuscript. We reconsidered that the radial defect in *shhb* mutants is induced by a decrease in Hedgehog signaling activity during both early and late development, not only during the late development. Fig. 7 highlights the summary of this suggestion. We discussed this in the first paragraph of the Discussion section as follows:
Page 12 Lines 319-335: "From these results, we suggest that the endochondral disc has two growth phases: an allometric growth during early development, and an isometric growth during late development (Fig. 7). During early development, the endochondral disc can change the ratio of the AP axis to the PD axis (AP-PD ratio), resulting in allometric growth (Fig. 7). Therefore, the number of pectoral fin radials is largely determined by the early growth of the endochondral disc. Conversely, the late growth of the endochondral disc may have an isometric AP-PD ratio. Although the number of radials is largely dependent on the early allometric growth of the endochondral disc, the Hedgehog signaling-promotive growth is buffered by *hhip* negative feedback. Both *shha* and *shhb* were expressed in the pectoral fins of zebrafish, while only *shha* was expressed in Japanese medaka (Fig. 3). Nevertheless, both fishes have the same number of radials in their pectoral fin skeletons. Although other differences, such as the activities of receptors or regulatory enhancers, may also regulate the strength of Hedgehog signaling in detail (Letelier et al., 2018; Lettice et al., 2017; Lopez-Rios et al., 2014), we suggest that the negative feedback effect of *hhip* buffers the different strengths of Hedgehog signaling to a certain level, generating four radials across teleosts."

Reviewer comment 1-4

4. The authors put a strong emphasis on the comparative/evolutionary context of their findings. Their phrasing in the manuscript is very careful and conjecturing because of course they do not actually show that hedgehog signaling is more active in basal fishes with elaborated fins. Although I thus think that their claims are appropriate, the evolutionary message will probably be the part of the paper that sticks with most readers (also because of the very nice homology drawings in Fig. 6). Therefore, if the authors have any data on basal species such as gar, sturgeon or shark indicating for instance lower levels of hhip expression they would be strongly encouraged to include these in the manuscript.

Response 1-4: Thank you for the critical comment. We wanted to perform the ISH of *hhip* using larva from basal species. However, it has proven too difficult for us to collect the necessary larva for these experiments. As an alternative approach to analyze basal fishes, we utilized open RNA-seq data from the pectoral fins of the brownbanded bamboo shark, bowfin and zebrafish. We then reanalyzed this data using the same pipeline. Our reanalyzed data (Fig. S10) suggests that the Hedgehog repression by *hhip* in the pectoral fins of zebrafish is stronger compared to that of basal species. We have included this data and corresponding text (see below) in the revised manuscript.
Page 9-10 Lines 252-271: "The *hhip* and *shh* analyses indicated that *hhip* expression during early development, when both duplicated *shh* genes were expressed, regulated pectoral fin elaboration in teleosts. However, it is unclear if early *hhip* expression is characteristic of teleosts. To compare *hhip* expression patterns in the pectoral fins of zebrafish with those of basal species, we used open RNA-seq data of pectoral fins in the brownbanded bamboo shark (Onimaru et al., 2021), bowfin (Thompson et al., 2021), and zebrafish (Kudoh et al., 2024). For this analysis, we reanalyzed the open RNA-seq data using the same pipeline (Patro et al., 2017). We selected *Hhip*-related genes (*Hhip, Ptch1, Ptch2,* and *Shh*), and *Actinodin* (*And*) genes for comparison of the developmental process (Fig. 5; Fig. S10). *And* encodes a structural protein specific to fin folds, a structure covering the outer edge of the endochondral disc and forming fin rays (Zhang et al., 2010). We used *And* genes to distinguish before and after the beginning of fin fold formation in each species (Fig. 5, the bottom row). *Ptch1* and *Ptch2* encode Hedgehog signaling receptors that compete with *Hhip*. For inter-species comparison of *Hhip* expression, we scaled the *Hhip* TPM by the sum of *Ptch1* and *Ptch2* TPM (Fig. S10, the top row). After fin fold formation, the scaled *Hhip* values for each species were almost the same. Before fin fold formation, however, the scaled value of zebrafish was higher than after fin fold formation, while those of the brownbanded bamboo shark and bowfin were lower than after fin fold formation. Taken together, the significant expression of *Hhip* before fin fold formation was characteristic of pectoral fin development in zebrafish."

Reviewer comment 1-5

© 2024. Published by The Company of Biologists under the terms of the Creative Commons Attribution License (https://creativecommons.org/licenses/by/4.0/).

5. Basal fishes also tend to have more caudal fin radials. Given the caudal fin phenotype in the hhip mutant the authors might want to include a line or two on this in the comparative paragraphs.

Response 1-5: We agree with the suggestion. The text has been revised, as follows:
Page 14 Lines 410-416: "However, the caudal fin skeletons in basal species have more hypurals than those in *hhip*$^{-/-}$ zebrafish (Cumplido et al., 2024). The increase in the number of ural vertebrae itself will be required to replicate the complete pattern of the caudal fin skeleton in basal species. Additional mutations modulating axial elongation (Aires et al., 2019; Cumplido et al., 2024; Kingsley et al., 2024) may generate sufficient ural vertebrae and hypurals to obtain a caudal fin skeleton of teleosts that is closer to that of basal species."

Reviewer comment 1-6
6. The authors might want to better highlight the effect on the anal fin in comparison to other hedgehog mutants. Its disappearance instead of being enhanced with increased hedgehog signaling is very interesting as it confirms prior work with gli3 and zrs mutant fishes which show a similar bizarre difference in hedgehog sensitivity between the anal and other fins. (But note that cichlids in contrast to other fish appear to have a ZPA in the anal fin (Höch et al. 2021) making the comparative context of these observations rather mysterious and difficult to understand.)

Response 1-6: We agree with the suggestion. The text has been revised, as follows:
Page 15 Lines 417-430: "In *hhip*$^{-/-}$ zebrafish, the dorsal fin radials increased, but the anal fin skeleton was completely absent (Fig. 1G-K). The opposite phenotypes in the median fin skeletons seem inexplicable, but the function of *hhip* on the dorsal-ventral regionalization in the somites may resolve the inconsistency. The *hhip* is expressed in the horizontal boundary cells of somites in teleosts and regulates the dorsal-ventral boundary of somites (Fig. S4A; Abe et al., 2019). The mutation of *hhip* in Japanese medaka expands the expression of *zic1*, a dorsal marker of somites, largely into the ventral part of somites (Abe et al., 2019). Since the somite cells contribute to the median fins as skeletal components, the median fin phenotypes presented here may be induced by the dorsalized somites, giving rise to an increase in dorsal fin components and a loss of the ventral ones that differentiate into anal fin skeletons. In median fin development, the effect of *Shh* within fin buds has been the focus so far (Dahn et al., 2007; Höch et al., 2021; Letelier et al., 2018; Letelier et al., 2021). We suggest that the regulation within somites is as important as that within fin buds for median fin development."

Response to Reviewer 2
Reviewer comment 2-1
While I fully appreciate the quality and novelty of this manuscript, this manuscript needs major improvements in the integration of all experimental results and scientifically accurate writing. Despite the excellence of each experimental result, they seem to not be functionally integrated well into the major goal of this paper: How are hhip, shh, and hox13 genes related to each other in the middle radial patterning? I also had a difficult time comprehending many writings due to scientific obscureness. The authors should thoroughly revise the text for scientific clarity. Finally, grammatical errors and typos are found throughout the manuscript. For further consideration of this manuscript, I highly recommend that the authors comprehensively improve the experimental scheme, the integration of all experiments, and text writing. See the detailed comments below.

Response 2-1: Thank you for the critical review and suggestion. The manuscript has been revised thoroughly.

Majors:
Reviewer comment 2-2
**In the paired fins of hhip knockout zebrafish, the second radial is divided and becomes multiple radials, recapitulating the morphological features of basal actinopterygians and chondrichthyans. As Hhip is a direct binding inhibitor of Hedgehog proteins, we would expect that hedgehog ligand knockout zebrafish also show defects around the second radial. However, Fig.5 exhibits that Shhb is functional in posterior pectoral fin morphogenesis. How do the authors explain this counter-intuitive difference in the affected areas between the pectoral fins of these knockout fish? Is the upregulation of Hedgehog signaling in hhip knockout fish caused by shha, not shhb? It would be great if the authors investigated active hedgehog signaling in developing fins, such as by ISH of the hedgehog signaling reporter gene (e.g., ptch). Also, is it possible to treat hhip mutant embryos*

© 2024. Published by The Company of Biologists under the terms of the Creative Commons Attribution License (https://creativecommons.org/licenses/by/4.0/).

with a chemical inhibitor of Hedgehog signaling and compare ptch expression patterns between wildtype, hhip mutant, hhip mutant treated by the inhibitor fish? It is highly recommended to explore the molecular underpinnings of the phenotypic discrepancy between hhip and shhb knockout zebrafish. The currently presented data, figures, and story are not integrated to substantiate the mechanisms that the authors assume.

Response 2-2: We agree with this suggestion and have examined the role of *hhip* mutation in pectoral fin buds. We have conducted the ISH of *ptch1* in WT and *hhip*$^{-/-}$ mutants (Fig. S4F, G). The data showed that Hedgehog signaling in *hhip* mutants was activated in the entire pectoral fin bud, especially in the anterior and posterior margins, while it was only expressed in the posterior margin in WT. Therefore, *hhip* mutation ectopically induced Hedgehog activity to the anterior region. We did not conduct chemical inhibitor experiments and the *ptch1* ISH because of the fragility of *hhip*$^{-/-}$ zebrafish. To resolve the phenotypic discrepancy between *hhip* and *shhb* knockout zebrafish, we speculate that the Hedgehog receptive region is not only the median region equivalent to the 2nd radial of WT, but also the posterior region equivalent to the 3rd and 4th radials of WT. From *ptch1* expression of WT, the median region can generate one radial without Hedgehog activity. From *ptch1* expression of *hhip*$^{-/-}$, Hedgehog signaling reaches the median region, resulting in the increase of radials. The *shhb* mutation decreased Hedgehog activity only in the posterior region, resulting in the defect in the 3rd and 4th radials. In summary, the median region can increase the number of radials in accordance of Hedgehog activity. However, the posterior region could not increase radials but needed a certain degree of Hedgehog activity to generate the normal 3rd and 4th radials. We have revised the related text as follows:
Page 13 Lines 364-378: "Lastly, our expression and mutation analyses highlighted the importance of the median region for pectoral fin elaboration. The *ptch1* of WT was expressed only in the posterior margin (Fig. S4F), while the *ptch1* expression reached both the median and posterior regions in *hhip*$^{-/-}$ zebrafish (Fig. S4G). Based on these expression patterns, Hedgehog signaling affected only the posterior region in *shhb*$^{-/-}$ zebrafish, exhibiting the fusion of posterior radials, and affected both the median and posterior regions in *hhip*$^{-/-}$ zebrafish, showing the excessive radials in the median region. We suggest that the median region is susceptible to Hedgehog signaling, which can increase the number of radials response to the signaling. On the other hand, the posterior region cannot increase radials but requires a certain degree of Hedgehog signaling to generate the normal 3rd and 4th radials. Although it has been believed that the pectoral fins of teleosts lost posterior radials branched from the metapterygium in the ancestral form and decreased to only four radials, we suggest that pectoral fin elaboration along the AP axis occurs through the reduction of radials in the median Hedgehog-susceptible region (Fig. 7, orange)."

Reviewer comment 2-3

**Hoxa13b and d13a expression patterns were monitored by EGFP knock-in zebrafish. These observations identified the three distinct domains with diverse hox expression patterns. While this finding could be informative to understand hhip mutant fish phenotype (and more generally, pectoral fin development, for sure), I'm wondering how this finding is better integrated into the main story of the manuscript. Is hox13 expression, particularly hoxa13b, regulated by the Hedgehog signaling? Is hoxa13b expression upregulated in hhip mutant fish? Ahn and Ho utilized Cyclopamine to reduce Hedgehog signaling and observed the disappearance of hoxa13a expression (Development, 2008). It is highly recommended that the expression level of hoxa13 in hhip mutant fish be analyzed.*

Response 2-3: As suggested, we crossed the *hhip*$^{-/-}$ and *hoxa13b*^{egfp}, and observed *hhip*$^{-/-}$ and *hoxa13b*$^{egfp/+}$ zebrafish (Fig. 6D-F). The data demonstrated that the *hhip* mutation expanded the EGFP expression of *hoxa13b*^{egfp}. Therefore, Hedgehog signaling may upregulate *hoxa13b*. This is consistent with the result of Cyclopamine treatment (Ahn and Ho, 2008). Integration of the *Hox* part to the main text has also been referred at Response 1-2. The data has been added (Fig. 6D-F) and the text has been amended as follows:
Page 10-11 Lines 293-301: "In *hhip*$^{-/-}$ zebrafish, *hoxa13b*^{egfp} was expressed in the distal margin, but EGFP was highly expressed in posterior cells compared to WT zebrafish (Fig. 6D, the arrowhead). Throughout the subdivision, the *hoxa13b*^{egfp} was expressed not only on the distal margin of the most posterior radials equivalent to the 3rd and 4th radials in WT zebrafish but also on the distal margin of the increased radials equivalent to the 2nd radial in WT zebrafish (Fig. 6E, F). These results suggest that the pectoral fin of zebrafish is regionalized by distinct patterns of *hoxa13* and *hoxd13* expression, and that the number of radials in *hhip* mutants increases in the median region where *hoxa13b* is expressed."

© 2024. Published by The Company of Biologists under the terms of the Creative Commons Attribution License (https://creativecommons.org/licenses/by/4.0/).

Reviewer comment 2-4
**In Fig. 6, the three colors were applied to indicate three different hox expression domains in pectoral fins. Although this is a novel attempt to comprehend developmental fin regionality, we don't know exactly which bones express which hox genes in catsharks, paddlefish, and bowfins. These color codes are misleading and should be presented as a hypothesis.*

Response 2-4: The Fig. 6 has been moved to supplemental data (Fig. S13A) and legend title has been revised to "The hypothesis of fin regionality and homology". Instead of the summary figure, we have designed a new Fig. 7 that summarizes the phenomena of pectoral fin development in zebrafish and mutants.

Minors:
Reviewer comment 2-5
Line 69-71: The current writing says, "Shh signaling reduces the number of radials in them," which is not what the authors meant, I suppose.

Response 2-5: Thank you, the text has been revised as follows:
Page 3 Lines 76-77: "suppressing Shh signaling in teleost pectoral fins moderately reduces the number of pectoral fin radials."

Reviewer comment 2-6
Line 113: "Regional-specific" should be "Region-specific".

Response 2-6: The text has been revised accordingly on Page 6 Line 141.

Reviewer comment 2-7
Line 125-127: I don't understand the writing and logic. This sentence needs to be more elaborated or clarified to explain how counterparts were identified between wildtype and mutant fish.

Response 2-7: The text has been revised as follows:
Page 7 Lines 154-164: "These observations indicated that the three featured subdivisions in *hhip*$^{-/-}$ zebrafish were equivalent to the 1st-3rd subdivisions in WT zebrafish. The 1st subdivision in both WT and *hhip*$^{-/-}$ zebrafish occurred much earlier than the other subdivisions (Fig. 2A, D), suggesting that these subdivisions are equivalent. The 2nd subdivision in WT forms the 1st proximal radial (PR1: Fig. 2F, dotted line), which has a curved shape different from the other proximal radials. The 2nd subdivision in *hhip*$^{-/-}$ zebrafish also formed the PR1 (Fig. 2C, dotted line). Therefore, the 2nd subdivision in *hhip*$^{-/-}$ zebrafish appears to be equivalent to the 2nd subdivision in WT (Fig. 2E, number 2). Since both WT and *hhip*$^{-/-}$ zebrafish formed two radials from the posterior half of the endochondral disc by a certain subdivision, the subdivision in *hhip*$^{-/-}$ zebrafish also appears to be equivalent to the 3rd subdivision in WT (Fig. 2F)."
Reviewer comment 2-8
Line 168: A hyphen is unnecessary.

Response 2-8: The text has been revised accordingly on Page 11 Line 309.

Reviewer comment 2-9
Line 172-173: Can this sentence be rephrased like "The high expression levels of hoxa13a/d13a in the distal region of posterior radials……"? I don't see the data that support the current writing, such as "The increased expression" or "with increasing proximity to the posterior region" in Fig. 3.

Response 2-9: The sentence has been deleted due to the revision of the entire manuscript.

Reviewer comment 2-10
Line 174: I guess the authors meant "region-specific expansion of endochondral radials" by "region-specific expansion".

Response 2-10: Thank you, the text has been revised accordingly.
Page 7 Lines 187-188: "the region-specific enlargement of the endochondral disc."

© 2024. Published by The Company of Biologists under the terms of the Creative Commons Attribution License (https://creativecommons.org/licenses/by/4.0/).

Reviewer comment 2-11
Line 198: "will have lost". Did the author mean "might have lost"?

Response 2-11: The text has been revised accordingly on Page 8 Line 212.

Reviewer comment 2-12
Line 222: "regulated" should be "regulates."

Response 2-12: The sentence has been deleted due to the revision of the entire manuscript.
Reviewer comment 2-13
Line 279: "these results" – did the author mean "current results" or "our results"? It's unclear what the authors refer to.

Response 2-13: It has been revised on Page 14 Line 399.

Reviewer comment 2-14
Line 292 should be like, "The first discrepancy is the repression mechanism of Hedgehog signaling."

Response 2-14: The sentence has been revised as follows:
Page 15 Lines 432-433: "The first difference is the suppression mechanism of Hedgehog signaling."

Reviewer comment 2-15
Line 301: Remove "in"

Response 2-15: The text has been revised at Page 15 Line 441.

Reviewer comment 2-16
Line 303: "regulated" should be "regulate(s)."

Response 2-16: The text has been revised on Page 15 Line 442.

Reviewer comment 2-17
Line 312: "shhb remains a paired fin enhancer…..". Shhb is not an enhancer.

Response 2-17: The text has been revised, as follows:
Page 15-16 Lines 451-452: " *the genomic region upstream of shhb in zebrafish contains a conserved paired fin enhancer.....*"
Reviewer comment 2-18
Line 326-327: If my understanding is correct, this sentence must be elaborated more for scientific accuracy. For example, does "augmented Hedgehog signaling" mean "augmented Hedgehog signaling from basal actinopterygians to teleosts" or what? What the authors meant is scientifically unclear.

Response 2-18: We have removed this during revision of the manuscript.

Response to Reviewer 3
Reviewer comment 3-1
Line 95 "In the pectoral fins of hhip-/- mutants, the pectoral girdle position was shifted laterally compared to that of WT zebrafish, which was similar to the position in chondrichthyans and basal actinopterygians (Fig. 1A, B" This is not clear from the results shown.

Response 3-1: In the submitted manuscript, we included a sentence to emphasize that the phenotype of the *hhip* mutant resembles that of chondrichthyans and basal actinopterygians. However, as pointed out by the reviewer, this point was not clearly supported by the results. Due to the challenge of obtaining data on the pectoral girdles of chondrichthyans or basal actinopterygians, we decided to delete the sentence "*which was similar to the position in chondrichthyans and basal actinopterygians* " in the revised version.

© 2024. Published by The Company of Biologists under the terms of the Creative Commons Attribution License (https://creativecommons.org/licenses/by/4.0/).

Reviewer comment 3-2
Line 114 "Fishes that retain the ancestral state of paired fins show an increase in radials along the AP axis during development (Davis et al., 2004; Mabee and Noordsy, 2004; Onimaru et al., 2016). To identify the regionality of these increases, we examined fin skeletons along the AP axis during the development of hhip-/- mutants (Fig. 2)."
The logic of this statement is unclear/muddled. The ancestral state is to have more radials and thus in teleost there has been a decrease of radials…

Response 3-2: As suggested, the entire manuscript has been thoroughly revised. The suggested sentence has been deleted.
Discussion:
Reviewer comment 3-3
The discussion sections contains some significant speculation and some misstatements/lack of clarity that requires some editing to correct.
Over interpretation. The results do not "explain the developmental change in the morphological evolution form the ancestral form to the teleost-specific derived form"
At best, they can support a potential developmental mechanism.

Response 3-3: The text has been revised accordingly.
Page 12 Lines 341-343: "support a potential developmental mechanism in the evolution of paired fins from the ancestral form to the teleost-specific derived form."

Reviewer comment 3-4
IT is not clear that the zf hhip mutant fins "largely correspond" to the endochondral bone formed in some chondrichthyans and basal actinopterygians.

Response 3-4: The text has been revised accordingly.
Page 13 Lines 350-352: "The subdivision order of the endochondral disc is similar between basal species and *hhip*$^{-/-}$ zebrafish, with the radials in the median region being the last to form (Fig. 2C)."

Minor
Reviewer comment 3-5
The title of the paper is vague and could perhaps be modifed to more explicitly state the core results of the manuscript.

Response 3-5: The title has been revised as "*Fin elaboration via anterior-posterior constraint by hhip on Hedgehog signaling in teleosts*".

Reviewer comment 3-6
No explanation is offered for the CRISPR induced gli3 mutant not replicating published zf gli3 mutants
Response 3-6: Previous studies on *gli3* in zebrafish have not reported phenotypes in fin radials (Devine et al., 2009; Wei et al., 2023). Especially, extended data (Figure 5) in one study (Wei et al., 2023) have shown that pectoral fin radials in *gli3* mutants appeared to have no abnormalities. Therefore, following sentence has been added:
Page 5 Lines 99-100: "Previous studies on *gli3* in zebrafish have also not reported any phenotypes in fin radials (Devine et al., 2009; Wei et al., 2023)."

Reviewer comment 3-7
223 Surprisingly, pelvic fin skeleton, including pelvic girdles, was absent in almost all shhb mutants (Fig. 5C).
This is not discussed further..why would an absence of fin be the predicted phenotype of absence of shh signalling in the fin?

Response 3-7: Thank you, the text has been revised accordingly:
Page 9 Lines 240-245: " Interestingly, in almost all *shhb* mutants, the entire pelvic fin skeleton, including pelvic girdle, was absent (Fig. 4C). A similar loss of the pelvic fin skeleton has been reported in Japanese medaka without the *shha* fin enhancer (Letelier et al., 2021). This indicated that the *shhb* mutation in zebrafish leads to the loss of Hedgehog signaling, resulting in the

© 2024. Published by The Company of Biologists under the terms of the Creative Commons Attribution License (https://creativecommons.org/licenses/by/4.0/).

absence of pelvic fin bud initiation."

Minor typographical
Reviewer comment 3-8
Line 280 "Although neither shha nor shhb expression were detected in the endochondral primordium of the caudal fin skeleton (Fig. S7A, B, E, F), Shh secreted by other organizers, such as the notochord, regulate hypural development (Hadzhiev et al.,2007)."
Incomplete second clause. Meaning is therefore unclear

Response 3-8: We have revised the second clause as follows.
Page 14 Lines 401-403: "...... , *Shh ligands secreted by other organizers like the notochord may regulate hypural development (Hadzhiev et al., 2007).*"

Reviewer comment 3-9
309 "has become a kind of gene desert" term "kind of" should be removed

Response 3-9: Thank you. The text has been revised accordingly. on Page 15 Line 450.

Reviewer comment 3-10
310 "located conserves the lmbr1 paralog" is unclear

Response 3-10: We have revised the text.
Page 15 Lines 448-449: "......, *the lmbr1 paralog has been conserved in the genomic region upstream of shha (Fig. S5).*"

Reviewer comment 3-11
133 "This developmental observation revealed an abnormality of the pelvic fin skeleton in hhip-/- mutants (Fig. 2G-L)" A new paragraph starts with this statement so it is unclear what "This developmental observation" relates to

Response 3-11: Thank you. The text has been revised accordingly.
Page 7 Lines 168-169: "The pelvic and caudal fin skeletons in *hhip*$^{-/-}$ zebrafish showed abnormalies during development (Fig. 2G-N)."

Reviewer comment 3-12
310 "considering that the expression pattern of Shh genes in paired fins of zebrafish and medaka, we speculate that zebrafish shhb remains a paired fin enhancer homologous to the ZRS of shha in the gene desert and achieves the fine-tuning regulation of Shh expression, while in medaka, the loss of one of the Shh genes simplifies such regulation"
Requires edits and modification to correct errors and to improve clarity

Response 3-12: We have revised the text as follows.
Page 15-16 Lines 450-455: "Considering that *shhb* in the paired fins of zebrafish was expressed for a similar duration as *shha* in medaka, it is possible that the genomic region upstream of *shhb* in zebrafish contains a conserved paired fin enhancer equivalent to the ZRS located within the *lmbr1* intron. On the other hand, the expression of *shha* in the paired fins of zebrafish has likely been modulated to be shorter than that of *shhb* in zebrafish and *shha* in Japanese medaka."
Reviewer comment 3-13
314"This diversity in Hedgehog signaling regulation" This has not actually been shown but is speculated on

Response 3-13: It has been removed due to the revision of the entire manuscript.

Second decision letter

MS ID#: DEVELOP/2023/202526

© 2024. Published by The Company of Biologists under the terms of the Creative Commons Attribution License (https://creativecommons.org/licenses/by/4.0/).

MS TITLE: Fin elaboration via anterior-posterior constraint by *hhip* on Hedgehog signaling in teleosts

AUTHORS: Yoshitaka Tanaka, Shun Okayama, Kohei Urakawa, Hidehiro Kudoh, Satoshi Ansai, Gembu Abe, and Koji Tamura

I have now received all the referees reports on the above manuscript, and have reached a decision. The referees' comments are appended below, or you can access them online: please go to BenchPress and click on the 'Manuscripts with Decisions' queue in the Author Area.

The overall evaluation is positive and we would like to publish a revised manuscript in Development, provided that the referees' comments can be satisfactorily addressed. The issue raised by Referee 1 regarding the readout for Hhip and the ratio to ptch1 and ptch2 should be addressed. In addition please address the minor comments listed by the referees. I would encourage you to carefully edit your manuscript for grammatical and typographical errors.

Please attend to all of the reviewers' comments in your revised manuscript and detail them in your point-by-point response. If you do not agree with any of their criticisms or suggestions explain clearly why this is so. If it would be helpful, you are welcome to contact us to discuss your revision in greater detail. Please send us a point-by-point response indicating your plans for addressing the refereesâ€™ comments, and we will look over this and provide further guidance.

Reviewer 1

Advance summary and potential significance to field

I am very much impressed by the revisions and this paper makes an important contribution to our insights into shh function and fin patterning as already pointed out in my first review. The authors convincingly demonstrate a role for buffering of shh activity by hhip that negatively influences the AP width of the fins and in addition they make a plausible case that this mechanism plays a role in the evolutionary modification of the teleosts body plan. The study contains a wealth of convincing experimental data on the mechanisms of shh patterning (shhb KO/KI, shh GFP KI in Medaka, hox GFP KI etc.) that go beyond this specific question and will provide valuable data and resources to the community working on the development and evolution of fins and limbs. Altogether, I am very enthusiastic about the current study and I am convinced that it will make an important and very visible contribution to Development.

Comments for the author

I have some rather minor comments (see end of this review) and, for sake of scientific accuracy also a more critical comment on some of the new data included.

I will start with this latter point.

- I applaud the authors for comparatively looking into hhip expression using published datasets for Bowfin and Shark. This analysis has been done quite meticulously and in the manuscript (Fig. 5) is presented to show that shark and Bowfin (which have ancestrally broader fins) have less buffering activity against shh activity.

However, instead of providing a simple readout for hhip (which can indeed be done using TPM) the ration between hhip expression and that of the two shh receptors ptch1 and ptch2 is provided. As I understand the reasoning behind this is that cellular readout of shh activity would be determined by the ratio between competing receptors and hhip. Although hypothetically this could be the case, I think that the current quantitative experimental understanding of shh signalling is far too limited to make such an assumption and to use this normalisation as the metric of choice for overall “shh activity". For instance, why not include the levels of shh itself in the equation? (BTW these are really low in shark, compare axes with the ray finned fish) After all it can also be reasoned that the ratio hhip to shh is what is important, or perhaps a triangular relationship between hhip, shh, and ptch? Or maybe something else. Looking at the raw data in Fig. S10 I do not think that the normalisation for ptch expression is required, or is scientifically an improvement for that matter.

© 2024. Published by The Company of Biologists under the terms of the Creative Commons Attribution License (https://creativecommons.org/licenses/by/4.0/).

Particularly for the Bowfin/Zebrafish comparison the values already seem convincing without normalisation. The shark might be more complicated, but it is important to consider that shark paired fin development may differ significantly from that of ray finned fishes in a number of ways (larger anterior aspect in form of the propterygium for instance, less reliance on shh - see also Onimaru et al. elife 2015 & 2021). When it comes to the question of the radial reduction in teleosts the shark is probably not the best out group to study as “ancestral model”. In contrast the Bowfin is the sister lineage to the teleosts and would be the model of choice for this question, thus this species should be seen as most conclusive in this context. Therefore, it would in my opinion be much cleaner if the authors would choose to present the Bowfin expression data in Fig. 5 and leverage for their main conclusions, while the ptch normalised data together with the shark could all go to the supplement in Fig S10.

-Then I have a number of minor comments:

Line 45; please mention already here that this transformation occurred in teleosts Line 63; “elaboration” should be “reduction” I guess Paragraph 228-249 For sake of completeness it would be good to comment briefly on how the fin phenotypes of sshb and published shha mutants compare.
Paragraph 273-309: I think it would be appropriate to integrate references to Sordino et al. Nature 1995 and Hawkins et al. Cell 2021 as refferral to the known AP pattern in zebrafish fins and the discussion concerning elaboration and the metapterygium, respectively.

Reviewer 2

Advance summary and potential significance to field

The authors conducted some additional experiments and comprehensively resolved my concerns. I fully appreciate the authors' effort to investigate ptch1 expression patterns in wild-type and hhip knockout embryos. The new experimental result (and the author's response to my comment) logically explains why only the second radial region is affected in hhip mutant embryos. Moreover, hhip-/-; hoxa13b:egfp data nicely bridges hhip mutant phenotype and hoxa13b expression pattern, which were dissociated in the original manuscript. Overall, the authors made significant improvements throughout the manuscript. Therefore, I would enthusiastically recommend this manuscript for publication in Development.

Tetsuya Nakamura

Comments for the author

NA

Reviewer 3

Advance summary and potential significance to field

There have been some revision made but further revisions are required in several sections to improve clarity

Comments for the author

Line 417 discussion

417. In hhip-/- zebrafish, the dorsal fin radials increased, but the anal fin skeleton was
418. completely absent (Fig. 1G-K). The opposite phenotypes in the median fin skeletons seem
419. inexplicable, but the function of hhip on the dorsal-ventral regionalization in the somites
420. may resolve the inconsistency. The hhip is expressed in the horizontal boundary cells of

© 2024. Published by The Company of Biologists under the terms of the Creative Commons Attribution License (https://creativecommons.org/licenses/by/4.0/).

421. somites in teleosts and regulates the dorsal-ventral boundary of somites (Fig. S4A; Abe
422. et al., 2019). The mutation of hhip in Japanese medaka expands the expression of zic1, a
423. dorsal marker of somites, largely into the ventral part of somites (Abe et al., 2019). Since
424. the somite cells contribute to the median fins as skeletal components, the median fin
425. phenotypes presented here may be induced by the dorsalized somites, giving rise to an
426. increase in dorsal fin components and a loss of the ventral ones that differentiate into anal
427. fin skeletons. In median fin development, the effect of Shh within fin buds has been the
428. focus so far (Dahn et al., 2007; Höch et al., 2021; Letelier et al., 2018; Letelier et al.,
429. 2021). We suggest that the regulation within somites is as important as that within fin
430. buds for median fin development.

This section is unclear.
The final statement is rather speculative, that has not been directly tested.

Minor typographical

L 34 Additionally, the gene expression reanalysis implied that -meaning is unclear

Line 38 Region-specific gene comparison
Meaning?

Line 120 hhip is significantly expressed in the entire pectoral fin bud at 48 hours post 121 fertilization (hpf) (Koudijs et al., 2005) -suggest remove “significantly”

Line 125 These results indicated that hhip works specifically during the early development of the paired fins in zebrafish Change to”suggests”

Line 192 However, expression patterns of Shh, a good candidate of Hedgehog ligands in paired appendages remain unclear in the late development of paired fins Statement unclear

502. Line 502 Standard length (in mm) is provided -Standard length of…?
503. Figure 1 K,L panels are anal fin but not clearly labelled as such to distinguish from median fins shown in G-J.

Second revision

Author response to reviewers' comments

Point-by-point response

Dear Editor:

Thank you for considering our manuscript. As per the suggestions, we have revised the manuscript, Fig. 5, and its legend. The reviewers’ comments have significantly helped us in improving the manuscript. We highly appreciate the constructive suggestions.

In addition to these points, we also found some typographical errors in the legends of the Supplemental Figures, particularly in Fig. S4, and have revised them. These revisions are indicated with red text in the legends of the Supplemental Figures.

Please see below our detailed point-by-point responses.

(Italics are reviewer and editor remarks to the authors)

© 2024. Published by The Company of Biologists under the terms of the Creative Commons Attribution License (https://creativecommons.org/licenses/by/4.0/).

Response to Reviewer 1

Reviewer comment 1-1

I applaud the authors for comparatively looking into hhip expression using published datasets for Bowfin and Shark. This analysis has been done quite meticulously and in the manuscript (Fig. 5) is presented to show that shark and Bowfin (which have ancestrally broader fins) have less buffering activity against shh activity.
However, instead of providing a simple readout for hhip (which can indeed be done using TPM) the ration between hhip expression and that of the two shh receptors ptch1 and ptch2 is provided. As I understand the reasoning behind this is that cellular readout of shh activity would be determined by the ratio between competing receptors and hhip. Although hypothetically this could be the case, I think that the current quantitative experimental understanding of shh signalling is far too limited to make such an assumption and to use this normalisation as the metric of choice for overall "shh activity". For instance, why not include the levels of shh itself in the equation? (BTW these are really low in shark, compare axes with the ray finned fish) After all it can also be reasoned that the ratio hhip to shh is what is important, or perhaps a triangular relationship between hhip, shh, and ptch? Or maybe something else. Looking at the raw data in Fig. S10, I do not think that the normalisation for ptch expression is required, or is scientifically an improvement for that matter. Particularly for the Bowfin/Zebrafish comparison the values already seem convincing without normalisation. The shark might be more complicated, but it is important to consider that shark paired fin development may differ significantly from that of ray finned fishes in a number of ways (larger anterior aspect in form of the propterygium for instance, less reliance on shh - see also Onimaru et al. elife 2015 & 2021). When it comes to the question of the radial reduction in teleosts, the shark is probably not the best out group to study as "ancestral model". In contrast the Bowfin is the sister lineage to the teleosts and would be the model of choice for this question, thus this species should be seen as most conclusive in this context. Therefore, it would in my opinion be much cleaner if the authors would choose to present the Bowfin expression data in Fig. 5 and leverage for their main conclusions, while the ptch normalised data together with the shark could all go to the supplement in Fig S10.

Response 1-1: We thank you for your constructive suggestion. We agree with your suggestion and have revised Fig. 5 to show the comparison of Hhip-related genes between bowfin and zebrafish. The expression data of brownbanded bamboo shark and the normalization value of *hhip* and *ptch* are in Fig. S10. We also have added the following sentence to the RNA-seq subsection in the Results section.

Page 10 Lines 269-270: *"The TPM of hhip in zebrafish was higher than that of bowfin, implying that hhip expression increases from basal actinopterygians to teleosts (Fig. 5)."*

Reviewer comment 1-2
Line 45; please mention already here that this transformation occurred in teleosts
Response 1-2: We have revised the text as follows.
Page 3 Lines 45-47: *"It is particularly noteworthy that pectoral fins, the anterior paired fins, have undergone morphological transformations in actinopterygians, resulting in a transition from their ancestral state to a derived form within the skeletal framework."*

Reviewer comment 1-3
Line 63; "elaboration" should be "reduction" I guess
Response 1-3: We wondered whether the text should be revised as "The reduction in pectoral fin radials" or should remain as "The elaboration of pectoral fins". Considering a previous sentence in our manuscript (Page 3 Lines 60-62; *"As a consequence of the radial constraint, teleosts have elaborated their pectoral fins into compact ones for speed adjustment, hovering, or horizontal directional changes"*), we decided to retain "elaboration". We hope this is understandable.

Reviewer comment 1-4

© 2024. Published by The Company of Biologists under the terms of the Creative Commons Attribution License (https://creativecommons.org/licenses/by/4.0/).

Paragraph 228-249 For sake of completeness it would be good to comment briefly on how the fin phenotypes of shhb and published shha mutants compare.

Response 1-4: We thank you for your constructive comment. As suggested, we have added sentences comparing the fin phenotypes of *shhb* and *shha* mutants, as shown below.

Page 9 Lines 240-243: *"Although the shhb$^{-/-}$ phenotype was mild compared to the severe phenotype of the shha complete loss mutants (Neumann et al., 1999), this data suggests that late Hedgehog signaling by shhb also contributes to the number of radials in the pectoral fin skeleton."*
Page 9 Lines 252-253: *"Taken together, shhb in zebrafish contributes to pectoral fin development along with shha and dominates pelvic fin development independently."*

Reviewer comment 1-5
Paragraph 273-309: I think it would be appropriate to integrate references to Sordino et al. Nature 1995 and Hawkins et al. Cell 2021 as referral to the known AP pattern in zebrafish fins and the discussion concerning elaboration and the metapterygium, respectively.
Response 1-5: We thank you for your suggestion. We added the following references and updated the reference list.
Page 10 Lines 282-285: *"Morphologically, the numerous radials in the pectoral fin of basal species are derived from the posterior region because they branch off from the metapterygium, the posterior tribasal bone (Gegenbaur, 1865; Hawkins et al., 2021; Mabee, 2000; Onimaru et al., 2015; Tanaka, 2018)."*
Page 10 Lines 290-292: *"These genes are expressed along the AP axis in some chondrichthyans, basal actinopterygians, and teleosts (Ahn and Ho, 2008; Davis et al., 2007; Freitas et al., 2007; Sordino et al., 1995; Tulenko et al., 2016)."*

Response to Reviewer 3
Reviewer comment 3-1
Line 417-430

"In hhip-/- zebrafish, the dorsal fin radials increased, but the anal fin skeleton was completely absent (Fig. 1G-K). The opposite phenotypes in the median fin skeletons seem inexplicable, but the function of hhip on the dorsal-ventral regionalization in the somites may resolve the inconsistency. The hhip is expressed in the horizontal boundary cells of somites in teleosts and regulates the dorsal-ventral boundary of somites (Fig. S4A; Abe et al., 2019). The mutation of hhip in Japanese medaka expands the expression of zic1, a dorsal marker of somites, largely into the ventral part of somites (Abe et al., 2019). Since the somite cells contribute to the median fins as skeletal components, the median fin phenotypes presented here may be induced by the dorsalized somites, giving rise to an increase in dorsal fin components and a loss of the ventral ones that differentiate into anal fin skeletons. In median fin development, the effect of Shh within fin buds has been the focus so far (Dahn et al., 2007; Höch et al., 2021; Letelier et al., 2018; Letelier et al., 2021). We suggest that the regulation within somites is as important as that within fin buds for median fin development."
This section is unclear. The final statement is rather speculative, that has not been directly tested.

Response 3-1: We agree with this comment and consider that the final sentences *("In median fin development, ...fin buds for median fin development.")* of this section are unclear. To clarify this section and tone down the final sentence, we have revised the sentences as follows.
Page 15 Lines 434-437: *"Possibly, the regulation within somites might be as important as that within fin buds for median fin development, although the effect of Shh within median fin buds has been the focus of studies so far (Dahn et al., 2007; Höch et al., 2021; Letelier et al., 2018; Letelier et al., 2021)."*

Reviewer comment 3-2
Line 36 "Additionally, the gene expression reanalysis implied that" meaning is unclear
Response 3-2: We have revised the text as follows.

Page 2 Lines 36: *"Additionally, the expression reanalysis of Hedgehog-related genes implied that…".*

© 2024. Published by The Company of Biologists under the terms of the Creative Commons Attribution License (https://creativecommons.org/licenses/by/4.0/).

Reviewer comment 3-3

Line 38 "Region-specific gene comparison" Meaning?

Response 3-3: We have revised the text as follows.

Page 2 Lines 38: *"Region-specific expression of Hox13 genes"*.

Reviewer comment 3-4
Line 120-121 "hhip is significantly expressed in the entire pectoral fin bud at 48 hours post fertilization (hpf) (Koudijs et al., 2005)" suggest remove "significantly"

Response 3-4: The text has been revised accordingly.

Page 6 Lines 120-121: "*hhip* is expressed in the entire pectoral fin bud at 48 hours post fertilization (hpf) (Koudijs et al., 2005)."

Reviewer comment 3-5
Line 125 "These results indicated that hhip works specifically during the early development of the paired fins in zebrafish"
Change to"suggests"
Response 3-5: The text has been revised as suggested.
Page 6 Lines 125-126: "These results suggest that *hhip* functions specifically during the early development of the paired fins in zebrafish."

Reviewer comment 3-6
Line 192 "However, expression patterns of Shh, a good candidate of Hedgehog ligands in paired appendages, remain unclear in the late development of paired fins"
Statement unclear

Response 3-6: We have revised the text as follows.

Page 8 Lines 191-194: *"In paired appendages of vertebrates, Shh is expressed as Hedgehog ligands antagonized by Hhip. However, the expression patterns of Shh in the late development of paired fins, when the endochondral disc grows and subdivides, remain unclear."*

Reviewer comment 3-7

Line 502 "Standard length (in mm) is provided"Standard length of...?

Response 3-7: We have revised this throughout the text as follows.

Page 20 Lines 497-498: *"The standard length* ***of individuals*** *(in mm) is shown"*.

Reviewer comment 3-8

Figure 1 K,L panels are anal fin but not clearly labelled as such to distinguish from median fins shown in G-J.

Response 3-8: We thank you for pointing out this issue. We believe the reviewer may have meant panels I and J, as these show the anal fin, while panels K and L display the caudal fin. We have revised the legend for panels I and J to clarify this. The revised legend is as follows.

Page 17 Lines 476-477: *"(I, J) Anal fin skeletons of hhip$^{-/-}$* ***(I)*** *and WT zebrafish* ***(J)"***.

© 2024. Published by The Company of Biologists under the terms of the Creative Commons Attribution License (https://creativecommons.org/licenses/by/4.0/).

Third decision letter

MS ID#: DEVELOP/2023/202526

MS TITLE: Fin elaboration via anterior-posterior constraint by *hhip* on Hedgehog signaling in teleosts

AUTHORS: Yoshitaka Tanaka, Shun Okayama, Kohei Urakawa, Hidehiro Kudoh, Satoshi Ansai, Gembu Abe, and Koji Tamura

I am happy to tell you that your manuscript has been accepted for publication in Development, pending our standard publication integrity checks.

© 2024. Published by The Company of Biologists under the terms of the Creative Commons Attribution License (https://creativecommons.org/licenses/by/4.0/).
